# High-fold Homogeneous Expansion Microscopy Reveals Ultrastructural Centrioles

**DOI:** 10.1021/acsnano.6c05501

**Published:** 2026-05-04

**Authors:** Wen-Qing Yang, Ting-Jui Ben Chang, Liang-Chen Pan, T. Tony Yang

**Affiliations:** † Department of Electrical Engineering, 33561 National Taiwan University, Taipei 10617, Taiwan; ‡ Graduate Institute of Biomedical Electronics and Bioinformatics, National Taiwan University, Taipei 10617, Taiwan

**Keywords:** ExM, expansion, high-fold, dSTORM, super-resolution, centrioles, ultrastructure

## Abstract

Super-resolution microscopy has transformed cellular imaging by enabling nanoscopic visualization of biomolecules. Expansion microscopy (ExM), which physically enlarges biological specimens, offers a complementary, optics-independent route to super-resolution. However, existing single-round high-fold ExM approaches have rarely been validated for their ability to preserve ultrastructural uniformity. Here, we present high-fold homogeneous expansion microscopy (hiHomoExM), a single-round ExM technique that achieves uniform ∼8–9*x* expansion while preserving cellular ultrastructure across diverse targets, including centrioles, nuclear pore complexes, and other organelles. hiHomoExM streamlines sample preparation and supports postexpansion labeling, allowing for both high labeling density and structural preservation. Furthermore, we extend the capability of hiHomoExM by integrating it into an iterative expansion framework to achieve even higher spatial resolution. Notably, coupling hiHomoEx with single-molecule localization microscopy (hiHomoEx-dSTORM) enables the resolution of previously elusive centriole ultrastructures, including the organization of CEP44, the microtubule-associated pattern of CCDC77, and the canonical 9-fold symmetry of SAS6. Together, hiHomoExM and hiHomoEx-dSTORM provide a robust and accessible platform for ∼2 nm-resolution characterization, bridging physical and optical super-resolution to advance cellular structural analysis.

## Graphical abstract

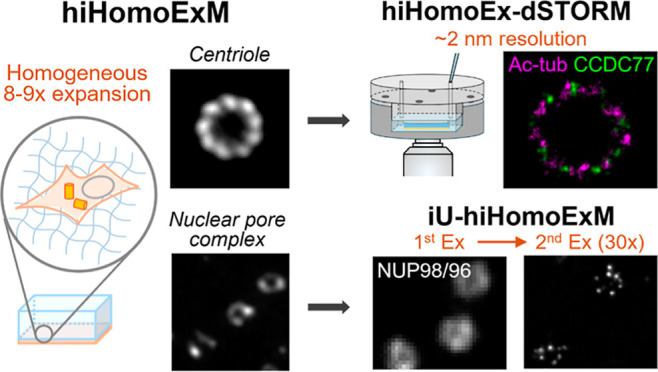

## Introduction

Super-resolution microscopy (SRM) has revolutionized cellular biology by enabling the identification and localization of biomolecules with nanometer precision. By surpassing the diffraction limit, techniques such as stimulated emission depletion microscopy (STED)^1^ and single-molecule localization microscopy (SMLM; e.g., (f)­PALM, (d)­STORM, (DNA-)­PAINT, and MINFLUX)^2–7^ extend spatial resolution to the nanoscale, advancing the field fluorescence nanoscopy. Beyond optical-based approaches, expansion microscopy (ExM)^8–10^ has emerged as a powerful super-resolution strategy that leverages sample preparation rather than optical manipulation. By physically expanding biological specimens, ExM enables super-resolved visualization of biomolecules using conventional microscopy. In particular, postlabeling ExM offers several advantages, including higher labeling density, reduced linkage error, and prevention of fluorophores from degradation typically observed in prelabeling protocols. Since ExM operates independently of optical-based SRM techniques, combining the two methods (Ex-SRM) enables the pursuit of molecular-level resolution. Among various Ex-SRM methods—such as Ex-STED,^11^ Ex-SOFI,^12,13^ Ex-SIM,^14^ and Ex-SRRF^15,16^ —Ex-SMLM^17–20^ exhibits the highest resolving potential, markedly advancing nanoscale biological research.

Over the past decade, numerous expansion strategies have been developed for diverse biological applications. These methods typically involve embedding specimens in a swellable hydrogel, followed by ideally uniform expansion of the gel after sample homogenization. The effective spatial resolution thus improves proportionally with the expansion factor. Modifying hydrogel chemistry, such as reducing cross-linker concentrations in acrylamide (AA)-based gel formulations or incorporating *N*,*N*-Dimethylacrylamide (DMAA), has enabled expansion factors to increase from the original 4-fold (4*x*)^8,10,21,22^ to approximately 10*x*.^13,23–25^ Iterative expansion techniques have further extended the expansion ceiling to over 20-fold,^26–28^ dramatically enhancing effective spatial resolution to levels comparable to SMLM, albeit with increased experimental complexity. Recently, DMAA-based gel chemistry modifications under deoxygenated conditions have enabled single-round expansion up to 20*x*, achieving performance comparable to iterative methods, as demonstrated by 20ExM.^29^ However, efforts to achieve higher expansion factors have paid limited attention to subcellular ultrastructural characterization. Although U-ExM (∼4*x*)^22^ has demonstrated ultrastructural preservation in centrioles—a compact organelle confined to a tiny volume and challenging to resolve using previous methods—high-fold ExM remains insufficiently validated and systematically explored.

To this end, we set out to develop a high-fold ExM method capable of preserving subcellular ultrastructure. Here, we present high-fold homogeneous expansion microscopy (hiHomoExM), which achieves uniform 8–9-fold expansion across gel, cells, and organelles while preserving ultrastructural details. This allows the specific visualization of centriolar chirality with conventional microscopy. Leveraging its simplicity in sample processing and gel handling, along with its ability to preserve molecular integrity, postlabeling hiHomoExM emerges as a promising platform for ultrastructure investigation in combination with optical-based SRM. By applying hiHomoEx with dSTORM, attaining an effective resolution of approximately 2 nm, we uncovered a previously uncharacterized, periodically stacked organization of CEP44 at the proximal region of the centriole. Our imaging also revealed the ultrastructural localization of CCDC77, which exhibits an evident pattern of linkage to microtubules. Furthermore, we successfully resolved the canonical 9-fold symmetry of SAS6, the cartwheel structure of the centriole. Together, hiHomoEx-dSTORM advances an promising imaging tool for exploring ultrastructural insights through optical approaches.

## Results and Discussion

### High-Fold Homogeneous Expansion Microscopy (hiHomoExM)

To facilitate ultrastructural investigation of protein complexes via sample expansion, we developed a strategy that targets a high expansion factor (EF) while uniformly preserving ultrastructural details (Figure 1a). Prioritizing accessibility for most laboratories, we designed the expansion protocol to be simple, featuring a single-round expansion, a streamlined experimental procedure, and easy-to-handle hydrogels made from commercially available materials. To assess its performance, we selected the human centriole as a reference due to its well-characterized dimensions (∼500 nm in length and ∼ 200 nm in width) and its extensive examination with both optical SRM and electron microscopy (EM).^19,30–38^ Additionally, we used U-ExM,^22^ a well-established expansion protocol with an EF of approximately 4, known for retaining ultrastructural information through homogeneous expansion at the nanometer scale, as a benchmark for comparison.

**Figure 1:**
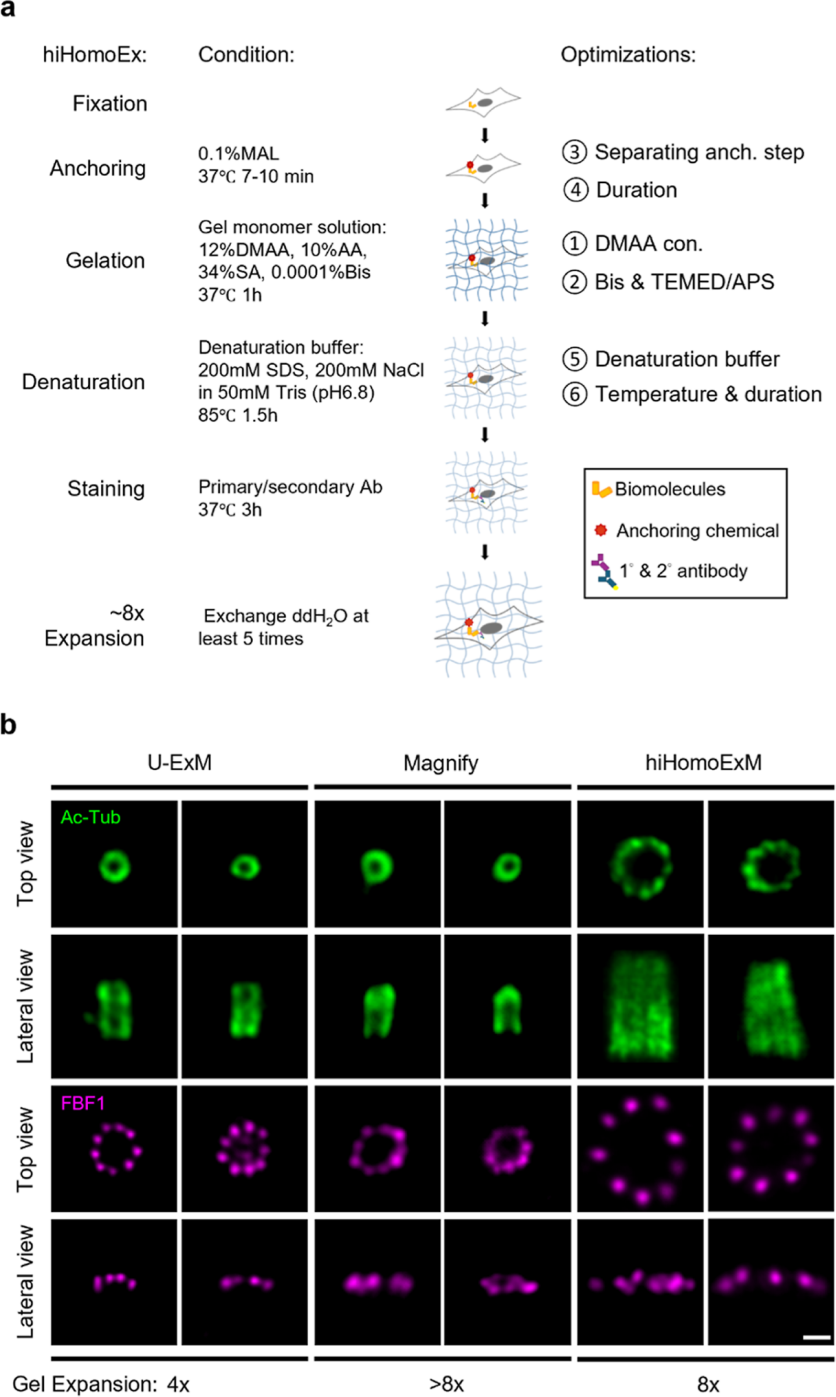
hiHomoExM workflow and comparative imaging of centriolar ultrastructure. (a) Schematic illustration of the hiHomoExM workflow, highlighting the optimized steps. (b) Representative images obtained using U-ExM, Magnify, and hiHomoExM for comparison. Centriolar proteins were immunolabeled with acetylated tubulin (Ac-tub, green; AF488) and the distal appendage protein FBF1 (magenta; CF568). Scale bar, 1 μm.

Given the need for greater expansion and easy gel handling, Magnify^13^ appeared as an ideal candidate. By adding a small amount of DMAA into the AA-based gel formation—a bifunctional monomer serving as the gel backbone and cross-linker—Magnify improves both gel rigidity and expansion factor. Although Magnify achieves an average gel expansion factor of ∼9.6*x* (Figure S1a,b), however, the resulting image of the centriole marker revealed a reduced cellular expansion, equivalent to the 4*x* expansion observed with U-ExM (Ac-Tub, Figure 1b). This discrepancy highlights a mismatch between the gel expansion factor and the actual expansion of cellular structures. A similar inconsistency was observed when using the distal appendage protein FBF1 as a reference (Figure 1b).

To address this issue, we developed a postlabeling expansion protocol that doubles the EF compared to U-ExM while maintaining ultrastructural integrity. This method, termed high-fold Homogeneous Expansion Microscopy (hiHomoExM), demonstrates a consistent expansion factor throughout the gel. The protocol leverages key advantages from various existing ExM approaches, delivering a streamlined workflow with targeted optimizations in the anchoring, gelation, and denaturation steps (Optimizations ①–⑥). Notably, with 8-fold improvements in resolution and labeling precision, hiHomoExM enables direct visualization of the canonical 9-fold symmetric arrangement of centriolar triplets using conventional epi-fluorescence microscopy (Figure 1b). A detailed description of the protocol and its development is provided in the following sections.

### Optimization of DMAA Concentration for Uniform Expansion

Building on the enhanced hydrogel rigidity achieved by incorporating DMAA into the AA-based gel recipe, we first evaluated how varying DMAA concentrations affect the uniformity of expansion across two key metrics: the macroscopic size of the hydrogel before and after expansion, and the nanoscale size of the centriole, benchmarked against normalized U-ExM measurements. We examined a range of DMAA concentrations from 2% to 12%. At 2%, the gel exhibited insufficient rigidity, while concentrations above 12% risked incomplete dissolution of sodium acrylate and compromised imaging signal quality (Figure S2). As DMAA concentration increased, we observed improved alignment between the macroscopic (gel) and nanoscopic (centriole) expansion factors (Figure 2a,b). These results indicate that higher DMAA concentrations enable more uniform expansion from the gel level down to subcellular structures, yielding a 6–7*x* expansion (Figure 2b). Normalization of the expanded centriole size to the average gel expansion factor further revealed structural consistency between U-ExM and the optimized hiHomoExM (Opt. ①) gel recipe containing 12% DMAA (Figure 2c). Moreover, the 12% DMAA formulation exhibited superior rigidity compared to the 4% used in the Magnify protocol (Figures 2b and S3a,b), enhancing both mechanical handling and compatibility with other optical-based SRM techniques. Taken together, these findings identify an optimal DMAA concentration (e.g., 12%) that ensures homogeneous expansion across macro-to nanoscale dimensions.

**Figure 2:**
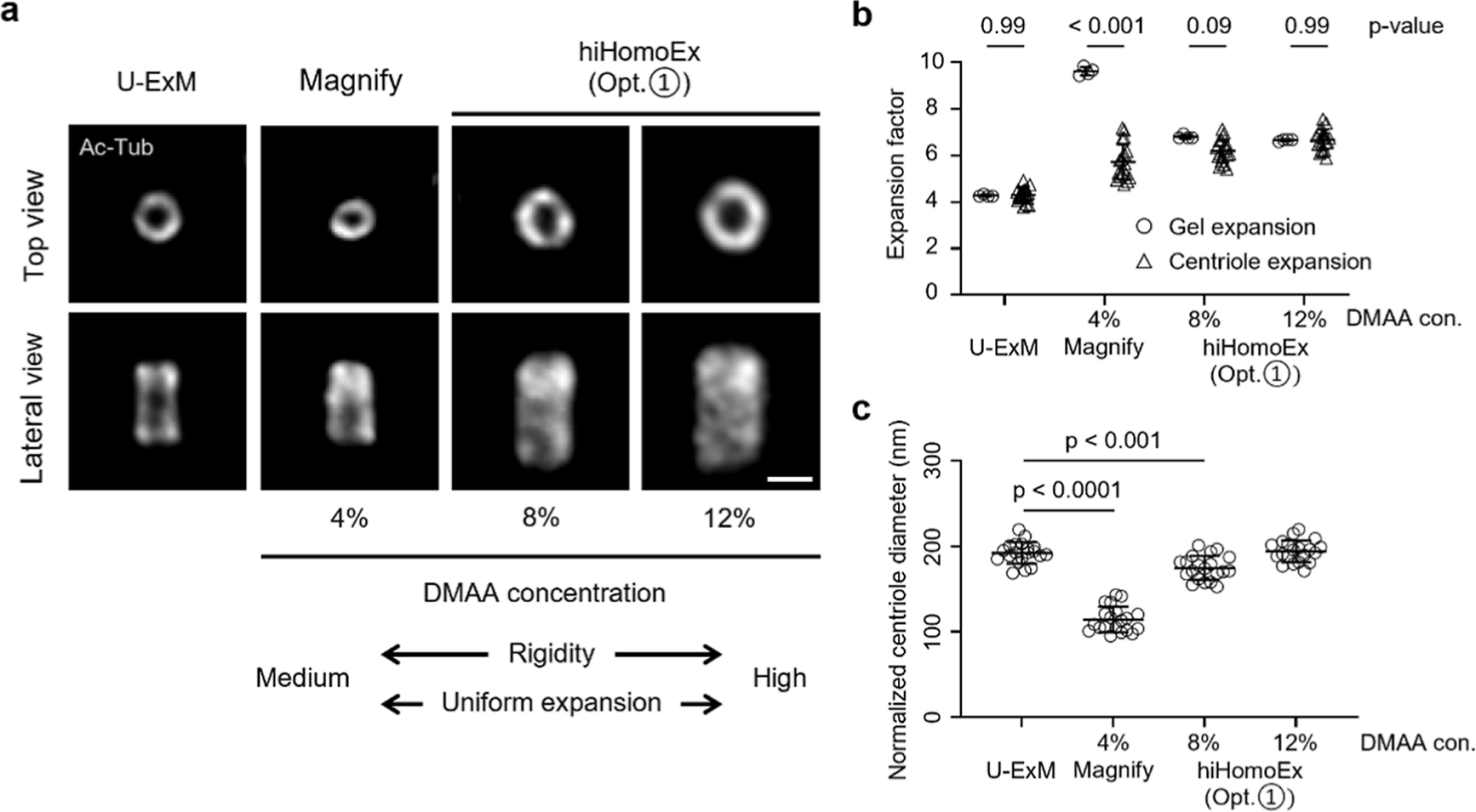
Optimization of DMAA concentration in hiHomoEx for refined gel and ultrastructure expansion. (a) Representative widefield images of centrioles stained for Ac-tub (gray; AF488). Samples were expanded using U-ExM, Magnify (4% DMAA), and hiHomoEx (Opt. ①; 8% and 12% DMAA). Scale bar, 1 μm. (b) Comparison of centriole expansion factors (EFs) and gel EFs across varying DMAA concentrations. Circles and triangles represent centriole EFs and gel EFs, respectively. Mean ± s.d. of gel EFs: 4.3 ± 0.03 for U-ExM (*n* = 4), 9.6 ± 0.16 for Magnify (4% DMAA, *n* = 4), 6.8 ± 0.07 for hiHomoEx (Opt. ①; 8% DMAA, *n* = 4), and 6.6 ± 0.03 for hiHomoEx (Opt. ①; 12% DMAA, *n* = 4). Mean ± s.d. of centriole EFs: 4.3 ± 0.27 for U-ExM, 5.7 ± 0.73 for Magnify (4% DMAA), 6.1 ± 0.48 for hiHomoEx (Opt. ①; 8% DMAA), and 6.6 ± 0.42 for hiHomoEx (Opt. ①; 12% DMAA). *n* = 20 centrioles per condition. Statistical analysis was performed using two-way ANOVA. (c) Normalized centriole diameters, calculated by dividing measured diameters by respective average gel EFs to estimate pre-expansion dimensions. Mean ± s.d. of normalized centriole diameters: 192.1 ± 12.3 nm for U-ExM, 114.2 ± 14.6 nm for Magnify (4% DMAA), 174.8 ± 13.7 nm for hiHomoEx (Opt. ①; 8% DMAA), and 194 ± 12.4 nm for hiHomoEx (Opt. ①; 12% DMAA). *n* = 20 centrioles per condition. Statistical analysis was performed using one-way ANOVA; *p*-values are shown for significant comparisons, and nonsignificant results are omitted.

### hiHomoExM Achieves Both Signal Retention and High Expansion Fidelity

With DMAA fixed at 12%, we investigated whether reducing the cross-linker Bis concentration could enhance the expansion factor, as reported in earlier studies without DMAA.^23,25^ Interestingly, we found a synergistic increase in expansion when low Bis concentrations were paired with reduced levels of the radical initiator/accelerator (APS/TEMED). The optimal condition of 0.0001% Bis and 0.15% APS/TEMED facilitated an approximately 8-fold expansion in blank gel (Figure 3a). Crucially, the expansion factor remained consistent from the macroscopic to nanoscopic scale, as validated by dSTORM imaging (Figure 3b,c). To assess ultrastructural expansion fidelity, we further determined the diameter ratio measured from two distinct centriolar structures (Figure 3b,d). Cross-validation with dSTORM and U-ExM confirmed that these optimized conditions preserved ultrastructural accuracy. Notably, the images showed a clear 2-fold increase in scaling for both the distal appendage marker (FBF1) and the centriole marker (Ac-Tub), relative to U-ExM (Figure 3b).

**Figure 3:**
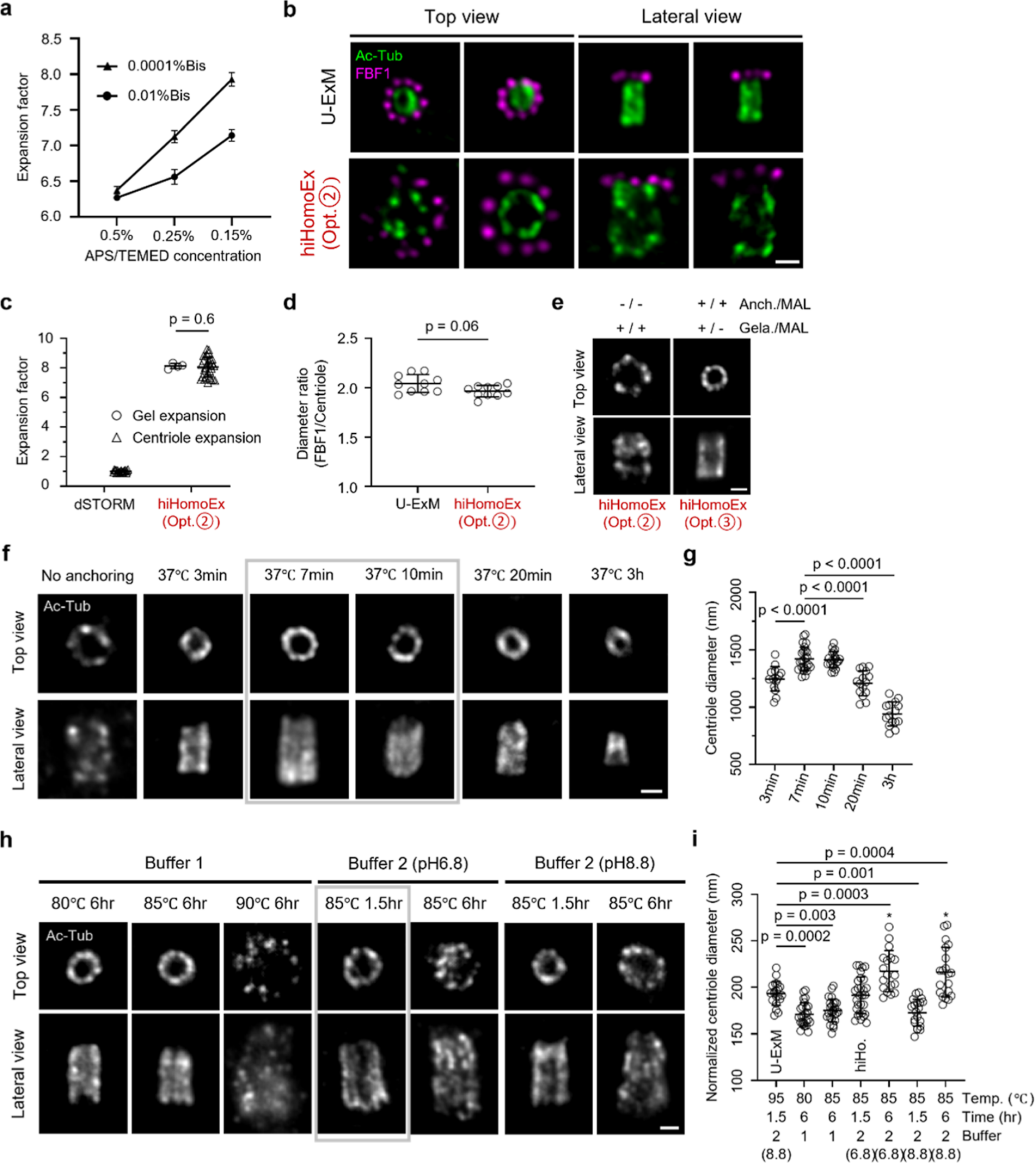
Systematic optimization of gel composition, anchoring, and denaturation conditions in hiHomoEx for homogeneous ultrastructure expansion. (a) Gel expansion factors measured under different concentrations of Bis and APS/TEMED. 0.01% and 0.0001% Bis were tested in combination with 0.5%, 0.25%, and 0.15% APS/TEMED, based on the hiHomoEx (Opt.①; 12% DMAA) gel formulation. Mean ± s.d. of gel expansion factors: 0.01% Bis: 6.3 ± 0.04 (0.5% APS/TEMED), 6.6 ± 0.08 (0.25% APS/TEMED), 7.1 ± 0.07 (0.15% APS/TEMED); 0.0001% Bis: 6.4 ± 0.04 (0.5% APS/TEMED), 7.1 ± 0.08 (0.25% APS/TEMED), 7.9 ± 0.04 (0.15% APS/TEMED). (b) Representative widefield images of centriolar proteins labeled with Ac-tub (green; AF488) and FBF1 (magenta; CF568) using U-ExM and hiHomoEx (Opt.②). (c,d) Evaluation of expansion homogeneity in hiHomoEx (Opt.②). (c) Comparative analysis of EFs for FBF1 and gel. Circles and triangles represent FBF1 and gel EFs, respectively. Mean ± s.d. of FBF1 EF: 1.0 (nonexpanded dSTORM, *n* = 19) and 8.0 ± 0.66 (hiHomoEx Opt.②, *n* = 30); mean ± s.d. of gel EF: 8.1 ± 0.16 (hiHomoEx Opt.②, *n* = 4). (d) Ratio of FBF1 ring diameter to centriole diameter measured from top-view images. Mean ± s.d. of ratios: 2.04 ± 0.08 (U-ExM, *n* = 10) and 1.97 ± 0.05 (hiHomoEx Opt.②, *n* = 10). Statistical analysis was performed using unpaired two-tailed *t* tests in (c,d). (e) Representative widefield images of centrioles labeled with Ac-tub (gray; AF488) under two anchoring conditions: (i) methacrolein included in the gelation step without a separate anchoring step (hiHomoEx Opt.②), and (ii) methacrolein applied in a separate anchoring step prior to gelation (hiHomoEx Opt.③). Anchr., anchoring; Gela., gelation; MAL, methacrolein. (f) Representative widefield images of centrioles labeled with Ac-tub (gray; AF488) under varying methacrolein anchoring durations. All samples were denatured at 80 °C for 6 h using denaturation buffer 1. (g) Quantification of centriole diameter from (f). Mean ± s.d. of diameters: 1246.6 ± 102.3 nm (3 min, *n* = 15), 1420.9 ± 101.9 nm (7 min, *n* = 25), 1414.2 ± 67.5 nm (10 min, *n* = 20), 1208.9 ± 104.8 nm (20 min, *n* = 15), 940.9 ± 101.9 nm (3h, *n* = 15). (h) Representative images of centrioles labeled with Ac-tub processed under distinct denaturation buffers, temperatures, and durations. All anchoring steps were performed at 37 °C for 7–10 min i Quantification of normalized centriole diameters from (h), using U-ExM as the reference. Mean ± s.d. of normalized diameters: 193.5 ± 12.4 nm (U-ExM, 95 °C 1.5 h, Buffer 2 pH 8.8, *n* = 20), 171.2 ± 12.3 nm (80 °C 6 h, Buffer 1, *n* = 25), 175.2 ± 12.1 nm (85 °C 6 h, Buffer 1, *n* = 26), 191.5 ± 19.2 nm (hiHo., 85 °C 1.5 h, Buffer 2 pH 6.8, *n* = 29), 217.4 ± 21.5 nm (85 °C 6 h, Buffer 2 pH 6.8, *n* = 18), 172.9 ± 14.0 nm (85 °C 1.5 h, Buffer 2 pH 8.8, *n* = 20), and 216.3 ± 26.1 nm (85 °C 6 h, Buffer 2 pH 8.8, *n* = 20). hiHo., hiHomoExM; *, indicates sample cracking artifacts. Statistical analysis was performed using one-way ANOVA in (g) and (i): p-values are shown for significant comparisons, and nonsignificant results are omitted. Scale bar, 1 μm (b, e, f, h).

With homogeneity successfully achieved, fluorescence signals for FBF1 were well-preserved; however, the discontinuous Ac-Tub signals indicated a need for further optimization. Although incorporating methacrolein—a small molecule that links biomolecules to the gel—into the gelation step eliminates the need for a separate anchoring process, our results showed improved structural integrity when anchoring and gelation were performed as distinct steps (Figure 3e). Further investigation into methacrolein anchoring durations revealed that centriole expansion closely matched gel expansion at an optimal incubation period. In contrast, prolonged anchoring times led to incomplete expansion, likely due to overfixation effects associated with methacrolein (Figure 3f,g).^39,40^

Lastly, we evaluated the effect of different denaturation conditions on sample expansion. Building upon a previously reported protocol (Buffer 1:10% (w/v) SDS, 8 M urea, 25 mM EDTA, 2x PBS, pH 7.5 at 80 °C for 6 h),^13^ we first increased the denaturation temperature. A modest improvement in the EF was observed at 85 °C, whereas treatment at 90 °C led to substantial signal loss (Figure 3h,i). We also tested an alternative denaturation buffer (Buffer 2:200 mM SDS, 200 mM NaCl, and 50 mM Tris)^22,23,26,41^ under two pH conditions and various incubation times at 85 °C. Notably, a shorter treatment duration at pH 6.8 produced optimal results, balancing expansion factor and fluorescence signal retention (Figure 3h,i). Collectively, hiHomoExM demonstrates optimized hydrogel composition, anchoring strategy, and denaturation conditions (see Methods), offering a robust and accessible approach for high-fold expansion microscopy.

### hiHomoExM Retains Centriole Architecture during Expansion

To validate the expansion homogeneity of the finalized hiHomoEx after a series of optimizations, we conducted a comprehensive evaluation from multiple perspectives. First, we evaluated homogeneity by comparing the expansion factor at both the macroscopic gel level and the protein level using the distal appendage protein FBF1. When benchmarked against the intact cellular structure imaged by dSTORM, hiHomoExM maintained a consistent expansion factor across both levels, whereas Magnify showed discrepancies between the two (Figure 4a,b). Notably, the scaled biological dimensions obtained with hiHomoExM closely matched the typical FBF1 diameter measured by dSTORM (Figure 4c). Second, to further confirm nanoscopic expansion uniformity across different centriole compartments, we calculated the ratio of the FBF1 (distal appendage) diameter to that of the centriole core (Ac-Tub). This ratio, as measured from hiHomoExM samples, accurately reflected centriole architecture and was comparable to U-ExM measurements, while Magnify yielded a significantly lower value (Figure 4d,e).

**Figure 4:**
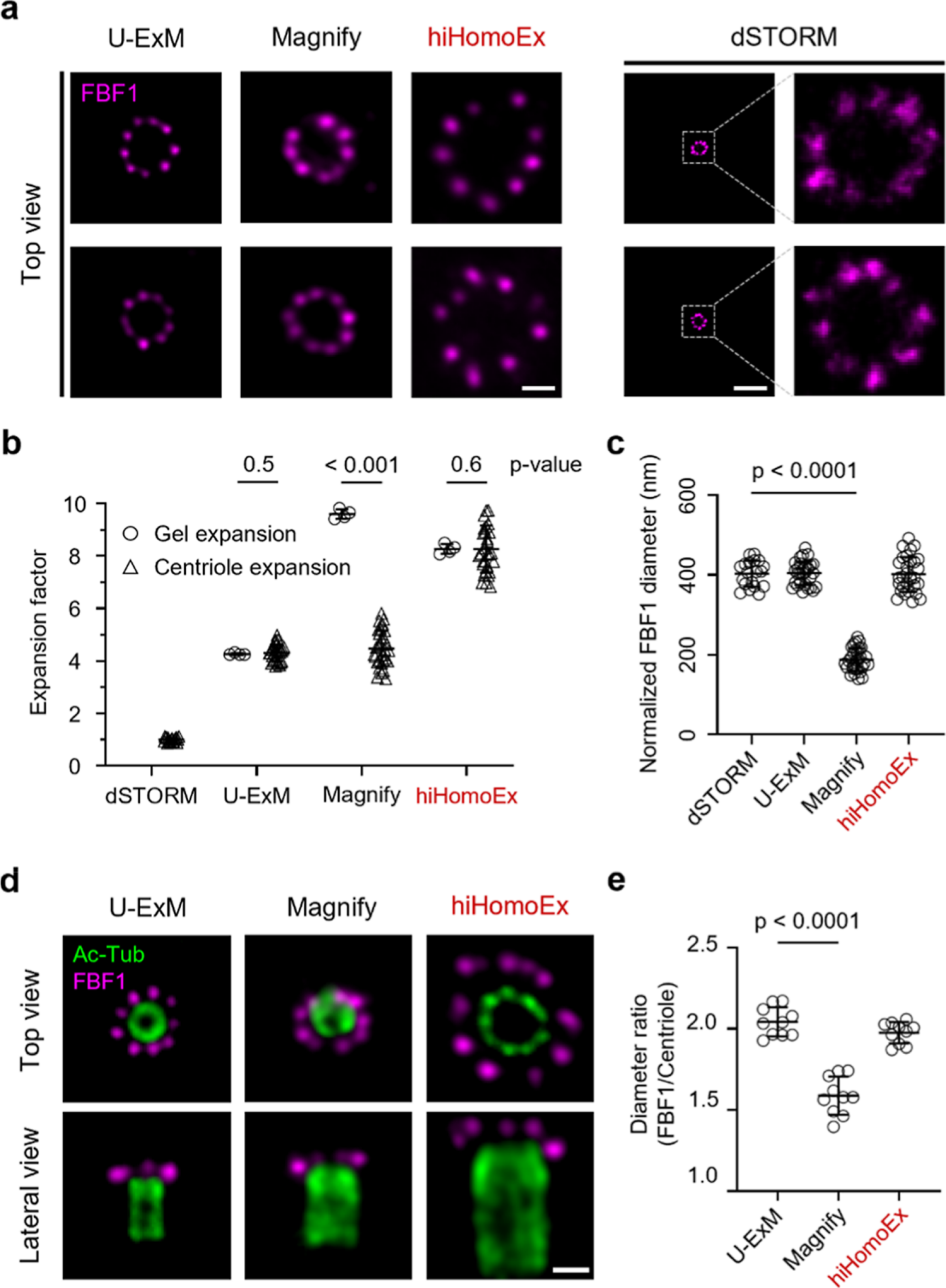
Comparative assessment of ultrastructural homogeneity among U-ExM, Magnify, hiHomoEx, and dSTORM. (a) Representative images of FBF1 (magenta; CF568) obtained with U-ExM, Magnify, hiHomoEx, and dSTORM imaging. (b) Expansion factors of gels and FBF1, using dSTORM as the nonexpanded reference. Mean ± s.d. of FBF1 EFs: 4.3 ± 0.31 (U-ExM, *n* = 30), 4.5 ± 0.67 (Magnify, *n* = 30), 8.3 ± 0.89 (hiHomoEx, *n* = 30), and 1 (dSTORM, *n* = 19). Mean ± s.d. of gel EFs: 4.3 ± 0.03 (U-ExM, *n* = 4), 9.6 ± 0.16 (Magnify, *n* = 4), and 8.3 ± 0.16 (hiHomoEx, *n* = 4). Statistical analysis was performed using two-way ANOVA. (c) FBF1 diameters normalized by the corresponding average gel EF to estimate pre-expansion dimensions (except for dSTORM). Mean ± s.d.: 404.5 ± 29.1 nm (U-ExM, *n* = 30), 188.1 ± 28.2 nm (Magnify, *n* = 30), 401.6 ± 43.2 nm (hiHomoEx, *n* = 30), and 403.2 ± 31.5 nm (dSTORM, *n* = 19). (d) Representative images of centriolar proteins stained for Ac-tub (green; Alexa Fluor 488) and FBF1 (magenta; CF568) via U-ExM, Magnify, and hiHomoEx expansion. (e) Ratio of FBF1 to centriole diameter measured from the top view, using U-ExM as the reference. Mean ± s.d.: 2.04 ± 0.08 (U-ExM, *n* = 10), 1.59 ± 0.11 (Magnify, *n* = 10), and 1.98 ± 0.06 (hiHomoEx, *n* = 10). Statistical analysis was performed using one-way ANOVA in (c) and (e). *P*-values are shown for significant comparisons. Scale bar, 1 μm (a, d).

To further confirm that hiHomoExM preserves uniform expansion, we quantified the radial symmetry of distal appendage proteins in unexpanded and expanded samples. The observed organization was consistent with protein-dependent variations reported in previous studies^37,42^ and recapitulated here using U-ExM (Figure S4). It reflects resolution-dependent differences in measured circularity (Figure S5), indicating that hiHomoExM preserves molecular architecture with fidelity approaching dSTORM resolution. We also examined centriole orientation, showing that well-preserved centriole structures can be observed across different viewing angles; however, near-perfect circularity is achieved only in a precisely axial view, while even slight tilting relative to the imaging plane can make the ring appear elliptical or less circular in projection (Figure S6). Together, these analyses substantiate that hiHomoExM achieves homogeneous expansion throughout the cell, while faithfully preserving the spatial architecture of molecular assemblies within distinct centriole compartments. As such, hiHomoExM features a powerful tool for investigating centriole ultrastructure.

### hiHomoExM and iU-hiHomoExM Reveals Diverse Subcellular Structures

To further demonstrate the broad utility of hiHomoExM, we evaluated its performance across multiple subcellular targets, including microtubules (α-tubulin), the Golgi apparatus (GM130), mitochondria (TOMM20), and the nuclear pore complex (NUP96) (Figures 5 and 6). Across these structures, hiHomoExM provided an ∼8–9*x* improvement in effective resolution compared to pre-expansion imaging, enabling visualization of structural features that were not discernible prior to expansion. In particular, bundled microtubule filaments and the TOMM20-labeled outer mitochondrial membrane were clearly resolved, demonstrating enhanced nanoscale structural detail (Figure 5a,c).

**Figure 5:**
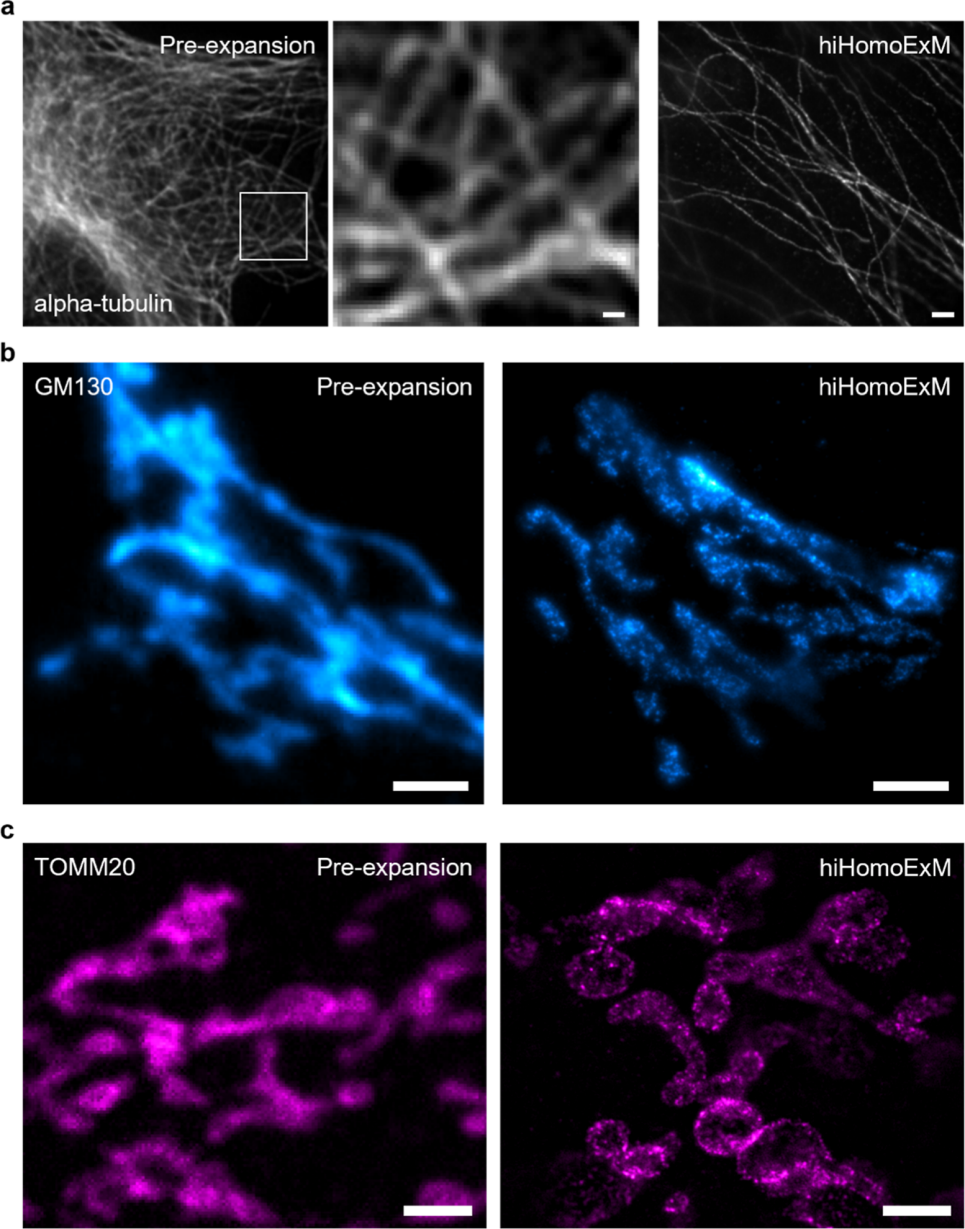
General applicability of hiHomoExM to multiple cellular structures. (a) Representative pre-expansion and hiHomoExM images of microtubules labeled with alpha-tubulin (gray; CF568); white box indicates the region shown in the pre-expansion zoom-in. Scale bar, 500 nm. (b) Representative pre-expansion and hiHomoExM images of the Golgi apparatus labeled with GM130 (cyan hot; AF647). Scale bar, 2 μm. (c) Representative pre-expansion and hiHomoExM images of the mitochondrial outer membrane labeled with TOMM20 (magenta hot; CF568). Scale bar, 2 μm. All scale bars are shown in biological scale.

**Figure 6:**
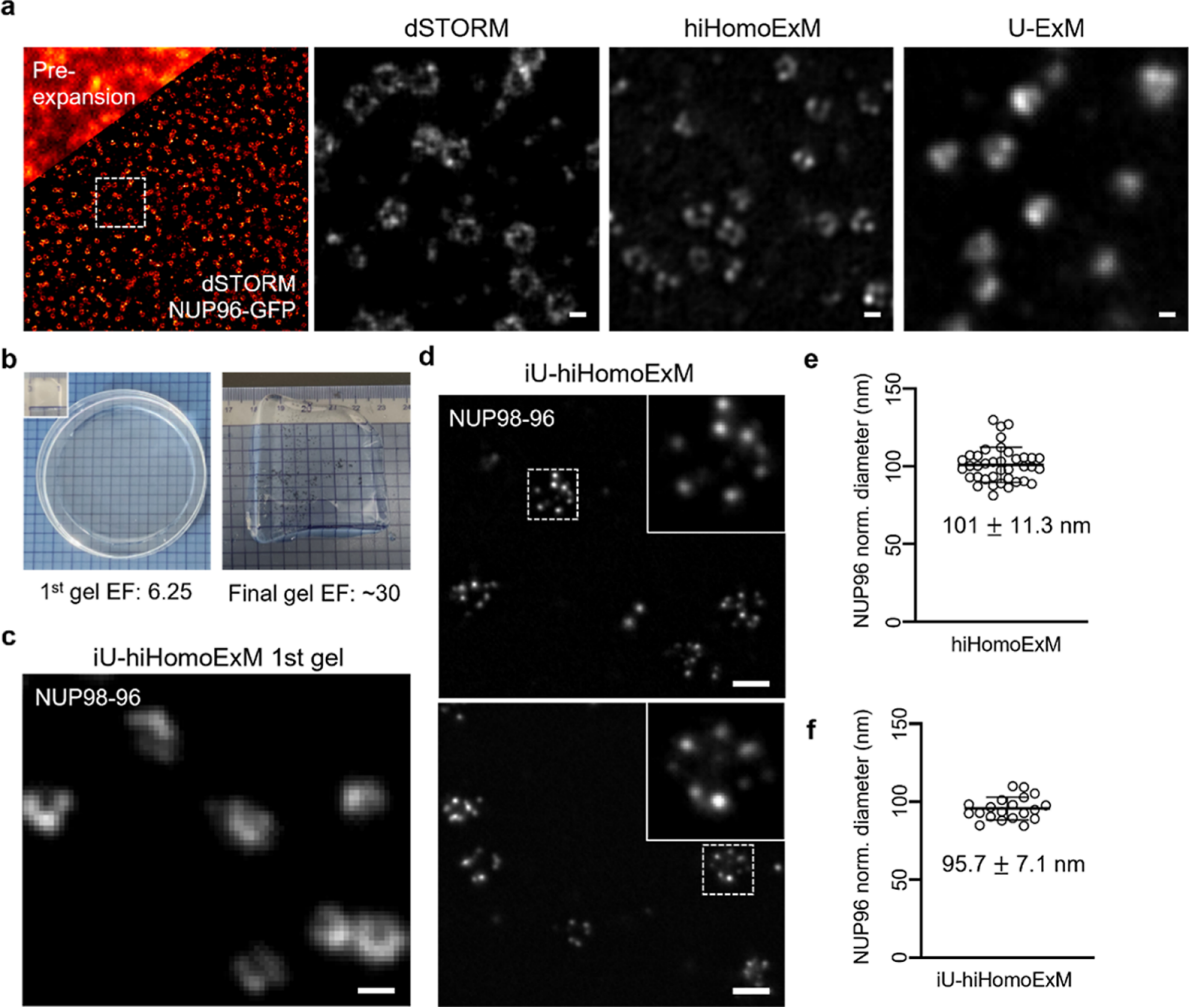
Homogeneous expansion of nuclear pore complexes and compatibility of hiHomoExM with iterative expansion. (a) Representative images of NUP96-labeled nuclear pore complexes (U2OS-NUP96-mEGFP cells; gray; CF568) acquired by pre-expansion imaging, dSTORM, hiHomoExM, and U-ExM; white dashed box indicates the region shown in the dSTORM zoom-in. Scale bar, 100 nm. (b) Images of the first gel and final gel from iU-hiHomoExM. (c) Representative images of NUP96 in the first gel of iU-hiHomoExM labeled with NUP98-96 (gray; CF568). Scale bar, 100 nm. d Representative images of NUP96 in iU-hiHomoExM labeled with NUP98-96 (gray; CF568); zoom-in images correspond to the white dashed box. Scale bar, 100 nm. (e) Quantification of normalized NUP96 diameters using hiHomoExM. Mean ± s.d. of normalized diameters: 101 ± 11.3 nm. (f) Quantification of normalized NUP96 diameters using iU-hiHomoExM. Mean ± s.d. of normalized diameters: 95.7 ± 7.1 nm.

We next assessed ultrastructural fidelity beyond centriole imaging using the nuclear pore complex component NUP96, a well-established reference for ultrastructural measurements. Comparable nuclear pore dimensions were observed across imaging modalities, including pre-expansion imaging, dSTORM, and U-ExM with hiHomoExM (Figure 6a). The normalized NUP96 diameter measured using hiHomoExM (∼101 nm) closely matched previously reported electron microscopy values (∼107 nm),^43,44^ indicating faithful preservation of native ultrastructural dimensions after expansion (Figure 6e). In addition, we demonstrated the compatibility of hiHomoExM with higher-order expansion strategies. By combining hiHomoExM with iterative U-ExM (iU-hiHomoExM), we achieved an overall expansion factor of approximately 30*x* while maintaining homogeneous expansion of both the gel and the embedded structures, with the normalized diameter of NUP96 measured at approximately 95 nm, consistent with measurements obtained using hiHomoExM alone (Figure 6b–d,f). Notably, this hybrid iU-hiHomoExM approach enabled visualization of NUP98-96-labeled nuclear pore complexes at very high expansion factors, clearly resolving the eight corner puncta of NUP96 (Figure 6d). Collectively, these results demonstrate that hiHomoExM enables high-resolution imaging across diverse subcellular structures while preserving ultrastructural organization and maintaining compatibility with iterative expansion strategies.

### hiHomoEx-dSTORM Elucidates Centriolar Ultrastructure

Finally, we employed hiHomoExM to investigate centriolar proteins spanning from the distal to the proximal end (Figure 7a–c). This approach enabled the visualization of multiple super-resolved structural features that are typically indistinguishable under conventional fluorescence microscopy, including ring-like structures, 9-fold symmetric, and centriolar chirality in top-view images, as well as distinct longitudinal protein distributions in lateral views (Figure 7a). To further assess the ultrastructural preservation, we performed dSTORM imaging on hiHomoEx-treated cells particularly prepared under a retention-optimized re-embedding condition (Figures 7d, S7 and S8a,b). We first examined the centriolar distal lumen protein C2CD3, comparing its organization between UEx-dSTORM and hiHomoEx-dSTORM. While UEx-dSTORM revealed the expected 9-fold symmetric pattern of C2CD3, hiHomoEx-dSTORM uncovered additional puncta within each C2CD3 cluster, suggesting finer substructural details (Figures 7e and S9). Next, we investigated the proximal end of the centriole, where many ultrastructural features have been previously characterized by electron microscopy.^45–47^ Specifically, we imaged CEP44 at the pinhead region, which bridges the cartwheel to the microtubule triplet.^48–50^ Our results exhibit that CEP44 is extensively localized in the longitudinal distribution (Figure 7a,b). Surprisingly, hiHomoEx-dSTORM imaging suggests that CEP44 arrangement may reflect the periodicity of the stacked pinhead structure (boxed, Figures 7f and S10a). Further quantitative Fourier analysis revealed an ∼26 nm spacing between CEP44 puncta, aligning with the characteristic pinhead periodicity observed in previous EM studies (Figure 7g,h).^45^ Furthermore, with the achieved molecular resolution, we successfully resolved the canonical 9-fold symmetry of the cartwheel, featuring a diameter of approximately 90 nm (SAS6-C terminus, Figures 7i and S10c). To gain further insight into centriole complexes in which microtubule triplets are interconnected, we performed two-color hiHomoEx-dSTORM imaging. Strikingly, the results revealed ultrastructural details of CCDC77 bridging the *C*-tubule of one triplet to the A-tubule of the neighboring triplet (Figures 7j and S10b). Together, these findings demonstrate the capability of hiHomoEx-dSTORM to visualize centriolar ultrastructure with high fidelity.

**Figure 7:**
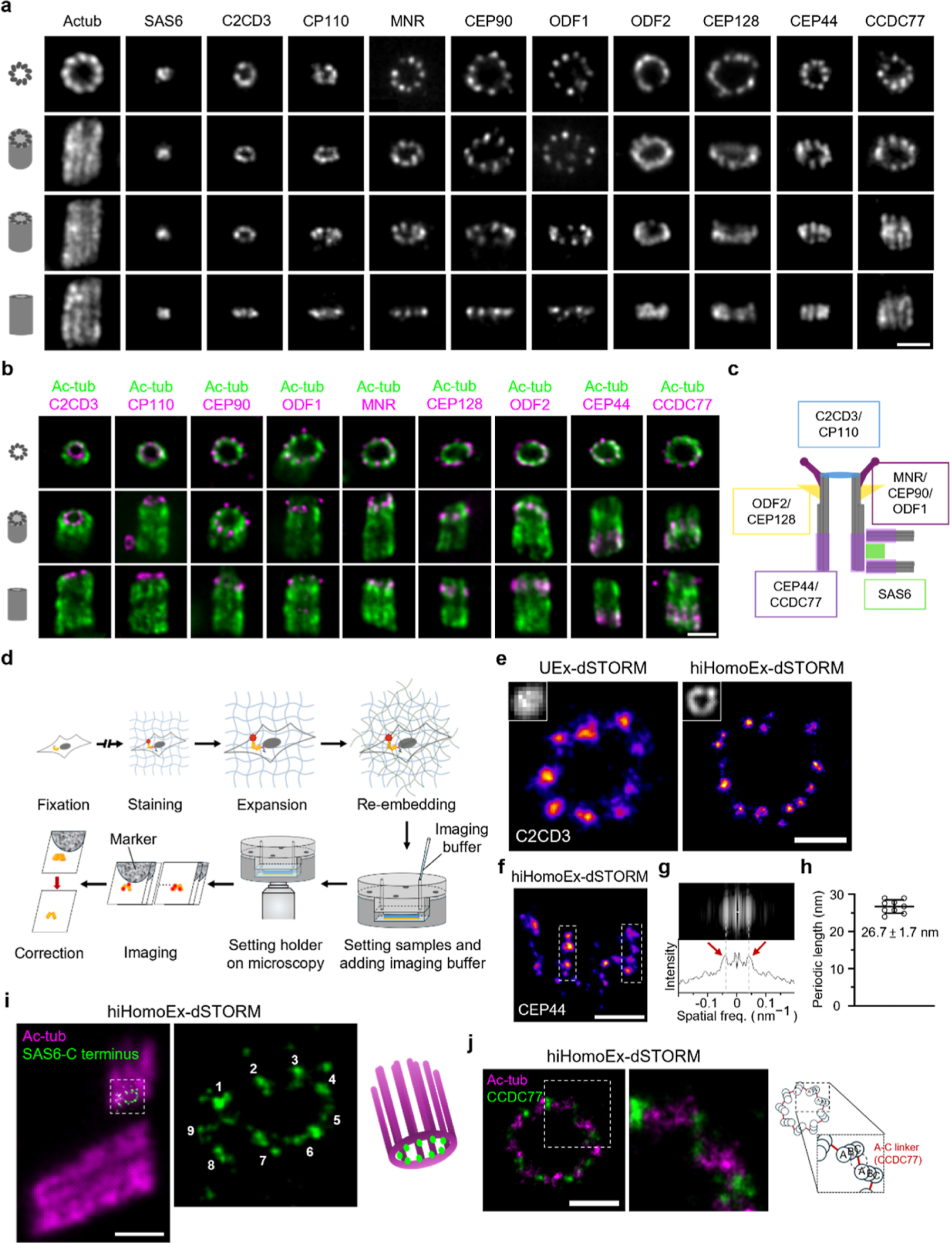
Unveiling nanoscopic details: hiHomoEx-dSTORM and widefield imaging reveal periodic patterns and structural organization of centriolar proteins. (a) hiHomoExM images of centriolar proteins captured across different orientations, from top view to lateral view, as indicated by the centriole cartoon. Scale bar, 250 nm. (b) Representative hiHomoExM two-color images of centriolar proteins (magenta; AF488) colabeled with acetylated α-tubulin (Ac-tub, green; CF568) as a structural reference, captured from top, tilted, and lateral views. Cartoons indicate the corresponding centriole orientations. Scale bar, 250 nm. (c) Schematic model depicting the structures shown in (a,b). (d) Schematic workflow of Ex-dSTORM, outlining key steps from expansion and re-embedding to dSTORM imaging. (e) Representative top-view images of C2CD3 acquired using UEx-dSTORM and hiHomoEx-dSTORM. Scale bar, 60 nm. (f–h) Periodicity analysis of CEP44. (f) Representative lateral-view hiHomoEx-dSTORM image of CEP44, with the white dashed box highlighting the periodic pattern. Scale bar, 100 nm. (g) Averaged stack of fast Fourier transforms (FFT) from CEP44 periodic patterns. Red arrows indicate the characteristic frequency of CEP44 periodicity. (h) Normalized periodic length of CEP44 determined from FFT analysis. Mean ± s.d.: 26.7 ± 1.7 nm (*N* = 9 centrioles, *n* = 54 intervals). (i) Representative hiHomoEx-dSTORM image of the SAS6 *C*-terminus (CF568; green) from a tilted view, merged with a widefield image of acetylated tubulin (Ac-tub, AF647; magenta). Scale bar, 200 nm. (j) Two-color hiHomoEx-dSTORM images of Ac-tub (AF647; magenta) and CCDC77 (CF568; green) in top view. Scale bar, 100 nm.

In this study, we developed hiHomoExM, a straightforward yet effective high-fold expansion microscopy method that preserves ultrastructure of protein complexes. By optimizing key parameters, including hydrogel formulation, molecular anchoring, and denaturation conditions, hiHomoExM achieves a homogeneous ∼8-fold expansion of the gel-specimen composite. Furthermore, the implementation of a postexpansion labeling strategy improves epitope accessibility, leading to enhanced labeling efficiency and increased labeling density (Figure S11). Importantly, it enables the visualization of ultrastructural features previously inaccessible with conventional 3–4*x* ExM techniques. These features include the canonical 9-fold symmetry of centriolar proteins and centriolar chirality (CEP44 and Ac-Tub; Figures 7a and S12a,b). Additionally, the improved mechanical stability of the gel makes it compatible with various imaging modalities and facilitates handling during sample processing. The gel composition of hiHomoExM also holds promise as a candidate for second-round expansion in iterative ExM, owing to its rigidity and high expansion factor (Figure 6b–d). Moreover, the overall sample preparation time is greatly reduced by incorporating a rapid methacrolein-based anchoring step, which can be completed within 10 min (Figure S13 and Table S1).

As a further advancement, the integration of hiHomoExM with dSTORM, implemented via an optimized re-embedding procedure (Figure S8a,b), unlocks the ability to resolve biomolecules separated by as little as 2–3 nm. This approach leverages both high spatial resolution and precise molecular labeling to reveal ultrastructural details. Using hiHomoEx-dSTORM, we visualized molecular features: intriguingly, CCDC77 appears as a linker connecting adjacent microtubule triplets, while CEP44 exhibits a periodic distribution at the proximal end (Figure 7f–h,j) previously inaccessible by conventional optical methods. These findings capitalize on the synergy between ultrastructural preservation and the high resolving power of hiHomoEx-dSTORM.

On the other hand, expansion factors may differ between the hydrogel and cellular components, among different organelles, or even across regions within the same organelle. While hiHomoExM was primarily validated using centrioles as an ultrastructural model, our results indicate that the method is broadly applicable to a wide range of subcellular structures. This suggests that the gel chemistry and processing conditions employed in hiHomoExM establish a stable and uniform expansion environment capable of preserving structural organization across diverse cellular architectures. Nevertheless, because organelles vary in protein composition, density, and ultrastructural organization, target-specific optimization of anchoring, denaturation, or staining conditions may still be necessary. Taken together, these findings position hiHomoExM as a generalizable framework for ultrastructural imaging, while retaining the flexibility to be adapted for different biological systems.

## Conclusions

Overall, hiHomoExM is a pragmatic and robust single-round high-fold expansion method that preserves the ultrastructural integrity of protein complexes. Beyond super-resolution imaging with conventional fluorescence microscopy, hiHomoExM further reveals molecular details when combined with dSTORM. This advancement in sample preparation and SMLM-based imaging offers a valuable tool for studying cellular biology at the ultrastructural level.

## Methods

### Reagents

Methyl alcohol (methanol, 15306121, Macron), Phosphate buffered saline (10X PBS, 70011044, Gibco), Paraformaldehyde (PFA, 16%, 15710, Electron Microscopy Sciences), Glutaraldehyde (GA, 8%, 16020, Electron Microscopy Sciences), Methacrolein (MAL, 96%, 043124.AA, Thermo Fisher Scientific), *N*,*N*-dimethylacrylamide (DMAA, 99%, 274135, Sigma-Aldrich), Sodium acrylate (SA, 97%, 408220, Sigma-Aldrich), Acrylamide (AA, 40%, A4058, Sigma-Aldrich), *N*,*N*′-methylenebis­(acrylamide) (Bis, 2%, M1533, Sigma-Aldrich), *N*,*N* ^′^-(1,2-Dihydroxyethylene)-bis­(acrylamide) (DHEBA, 98%, D2864, TCI Chemical), Acrylamide/Bis-acrylamide (30%, 29:1, 1610156, Bio-Rad), *N*,*N*,*N*′,*N*′-Tetramethylethylenediamine (TEMED, 1610801, Bio-Rad), Ammonium persulfate (APS, 1610700, Bio-Rad), Sodium dodecyl sulfate (SDS, 0227, VWR Life science), Sodium chloride (NaCl, 31434, Sigma-Aldrich), Ethylenediaminetetraacetic acid disodium salt (EDTA, 0.5M, E7889, Sigma-Aldrich), Urea (U5378, Sigma-Aldrich), Tris (1.5M, pH 8.8, J831, VWR Life science), Tris (0.5M, pH 6.8, J832, VWR Life science), Bovine serum albumin (BSA, A9647, Sigma-Aldrich), Tween 20 (P137, Sigma-Aldrich), Triton X-100 (T8787, Sigma-Aldrich).

### Antibodies

Comprehensive information on the primary antibodies used in this study is provided in the Table S2. The second antibodies used in this study were Alexa Fluor 647 (1/100 dilution, antimouse A31571, antirabbit A31573, antigoat A-21447; Thermo Fisher Scientific), CF568 (1/100 dilution, antimouse 20105, antirabbit 20098; Biotium), Alexa Fluor 488 (1/100 dilution, antimouse A21202; Thermo Fisher Scientific).

### Cell Culture

Human retinal pigment epithelial cells (hTERT RPE-1, ATCC-CRL-4000) were cultured in Dulbecco’s modified Eagle’s medium (DMEM)/F-12 and HEPES (1:1; Gibco, Thermo Fisher Scientific, 11330-032) at 37 °C in a humidified 5% CO_2_ incubator, supplemented with 10% fetal bovine serum (FBS, Hyclone, SH3010903), sodium bicarbonate (NaHCO3, S6014, Sigma-Aldrich), and 1% penicillin–streptomycin. U2OS-CRISPR-NUP96-mEGFP cells (Cytion, 300174) were cultured in Dulbecco’s modified Eagle’s medium (DMEM) with GlutaMAX (Gibco, Thermo Fisher Scientific, 10566-016) at 37 °C in a humidified 5% CO_2_ incubator, supplemented with 10% fetal bovine serum and 1% penicillin–streptomycin. Both cell lines were seeded onto poly-l-lysine coated 12 mm coverslips for expansion microscopy and 18 mm coverslips for dSTORM or pre-expansion imaging. Cells were then fixed using fixation conditions appropriate for the target proteins and imaging modality (Table S3).

### hiHomoEx

After fixation, cells with coverslips were anchored with 0.1% (v/v) methacrolein at 37 °C for 7–10 min. Next, aspirating anchoring solution and samples were washed two to three times (5 min for each) with 1x PBS. Each coverslip was then incubated with gel monomer solution (12% (v/v) DMAA, 34% (w/v) SA, 10% (w/v) AA, 0.0001% (w/v) Bis, 1% (w/v) NaCl and 1*x* PBS). APS and TEMED were both added to final concentrations of 0.15% (w/v), and gelation was carried out at 37 °C for 1 h. Following the gelation, coverslips with hydrogel were placed in fresh denaturation buffer (200 mM SDS, 200 mM NaCl in 50 mM Tris (pH6.8)) at RT until hydrogels detached from coverslips. Hydrogels were then incubated in fresh denaturation buffer at 85 °C for 1.5 h. After denaturation, the hydrogels were washed with 1*x* PBS three times at RT and expanded in fresh ddH_2_O at least five times (20 min for each) with gentle shaking until the hydrogels were fully expanded and then incubated overnight in ddH_2_O. The overall procedure is summarized in Table S3. (Note: Different brands or batch numbers of sodium acrylate may slightly affect the optimal conditions for the anchoring and denaturation steps.)

### U-ExM

After fixation, coverslip samples were incubated in perfusion solution (1.4% PFA and 2% AA) at 37 °C for 5 h. Next, aspirating perfusion solution and samples were washed two to three times (5 min for each) with 1x PBS. Then, incubating each coverslip with U-ExM monomer solution (19% (w/w) SA, 10%(w/w) AA, 0.1%(w/w) Bis in PBS) and added the chemicals APS and TEMED to a final concentration of 0.5% (w/v) APS and 0.5% TEMED (w/v) in a humidified chamber at 37 °C for 1 h. Following the gelation, coverslips with hydrogel were placed in fresh denaturation buffer (200 mM SDS, 200 mM NaCl in 50 mM Tris (pH8.8)) at RT until hydrogels detached from coverslips. Hydrogels were then incubated in fresh denaturation buffer at 95 °C for 1.5 h with shaking. After denaturation, the hydrogels were washed with 1*x* PBS three times at RT and expanded in fresh ddH_2_O at least three times (20 min for each) with gentle shaking until the hydrogels were fully expanded and then incubated overnight in ddH_2_O.

### Magnify

After fixation, incubating each coverslip with Magnify monomer solution (4% (v/v) DMAA, 34% (w/v) SA, 10% (w/v) AA, 0.01% (w/v) Bis, 1% (w/v) NaCl and 1*x* PBS) and added the chemicals APS, TEMED, and methacrolein to a final concentration of 0.25% (w/v) APS, 0.25% (w/v) TEMED, and 0.1% (v/v) methacrolein overnight in a humidified chamber at 37 °C. Following the gelation, coverslips with hydrogel were placed in fresh denaturation buffer (10% (w/v) SDS, 8 M Urea, 25 mM EDTA, 2*x* PBS, pH 7.5) at RT until hydrogels detached from coverslips. Hydrogels were then incubated in fresh denaturation buffer at 80 °C for 6 h with shaking. After denaturation, the hydrogels were washed with 1*x* PBS three times at RT and expanded in fresh ddH_2_O at least five times (20 min for each) with gentle shaking until the hydrogels were fully expanded and then incubated overnight in ddH_2_O.

### iU-hiHomoExM

Samples were initially treated with 0.1% methacrolein at 37 °C for 7–10 min to anchor cellular components. Coverslips were then immersed in first gel solution composed of 10% (w/v) AA, 19% (w/w) SA, 0.1% (w/v) DHEBA, and 0.25% (w/v) APS/TEMED. Gelation was performed on ice for 15 min followed by incubation at 37 °C for 45 min. The hydrogels were denatured in a buffer containing 200 mM SDS, 200 mM NaCl, and 50 mM Tris (pH 6.8) at 85 °C for 1.5 h, and subsequently expanded by repeated immersion in ddH_2_O.

For intermediate immunolabeling, the expanded gels were equilibrated in 1x PBS and incubated sequentially with primary and secondary antibodies at 37 °C for 3 h each. Following staining, gels were re-expanded in ddH_2_O. To stabilize the structure, the gels were embedded in a neutral polymer solution (10% (w/v) AA, 0.05% (w/v) DHEBA, 0.1% (w/v) APS/TEMED) via three successive incubations of 10 min each on ice with gentle agitation, and polymerization was carried out at 37 °C for 1 h in a humidified environment.

For reanchoring, hydrogels were incubated in a solution containing 1.4% PFA and 2% AA at 37 °C for 3 h, followed by washing in 1x PBS for 30 min. Gels were then equilibrated in a second gel solution (12% (v/v) DMAA, 34% (w/v) SA, 10% (w/v) AA, 0.0001% (w/v) Bis, 1% (w/v) NaCl, 1*x* PBS, and 0.1% (w/v) APS/TEMED) through three 10 min incubations on ice with gentle shaking. Excess solution was removed and gels were polymerized at 37 °C for 1 h in a humid chamber. The original and neutral gels were subsequently dissolved by treatment with 200 mM NaOH for 1 h at room temperature with agitation, followed by thorough washing in 1*x* PBS until neutral pH was restored. Final expansion was achieved by immersion in ddH_2_O.

For postexpansion labeling, gels were first shrunk in 1*x* PBS, then incubated with primary antibodies for at least 12 h at 37 °C, followed by secondary antibody incubation for 6 h at 37 °C. Gels were washed three times with 0.1% PBS-Tween20 (30 min per wash) and re-expanded in ddH_2_O prior to imaging.

### Pre Labeling Immunostaining

After fixation, coverslips with samples were washed three times with 0.1% PBS-Triton X-100 for 5 min each, followed by two washes with 1*x* PBS. Samples were then transferred into a staining chamber under a humidified environment. Cells were permeabilized with 0.1% PBS-Triton X-100 for 10 min at room temperature, followed by blocking of nonspecific antibody binding in 3% BSA prepared in PBS-Triton X-100 for 30 min at room temperature.

For immunostaining, samples were incubated with primary antibodies diluted in 3% BSA/PBS-Triton X-100 for 1 h at room temperature, followed by 5–10 washes with 0.1% PBS-Triton X-100. Secondary antibody incubation and washing were carried out using the same conditions as for the primary antibodies. Finally, samples were stored in 1*x* PBS until further use.

### Post Labeling Immunostaining

Before immunostaining, hydrogels were transferred from ddH_2_O to 1*x* PBS and kept them overnight, then cut hydrogels into an appropriate size for staining. Immunostaining was first incubated with primary antibodies diluted in 2% BSA/PBS at 37 °C for 3h or at RT for overnight in the Eppendorf with gentle shaking. The samples were then washed three times with 0.1% PBS-Tween 20 and twice with 1*x* PBS for 10 min each. Next, samples were carried out labeling with secondary antibodies diluted in 2% BSA/PBS at 37 °C for 3h with gentle shaking followed by the same washing steps. After immunostaining, the hydrogels were exchanged at least three times in ddH_2_O until fully they expanded.

### Re-embedding of hiHomoEx Expanded Hydrogels

Prior to Ex-dSTORM imaging, the expanded hydrogels need to be re-embedded in a neutral acrylamide gel for maintaining the size of hydrogels in imaging buffer while dSTORM imaging (Figure S7). First, the expanded hydrogels were immersed in fresh re-embedding solution (10% (w/w) AA, 0.15% (w/w) Bis, 0.025% (w/w) TEMED, 0.025% (w/w) APS in ddH_2_O) twice for 30 min each time at RT with gentle shaking. Before polymerization, the hydrogels need to use Kimwipe to remove the remaining re-embedding solution completely. In polymerization process, the hydrogels were then transferred to re-embedding chamber with two untreated coverslips covering between them and incubated them in a nitrogen-filled humidified box at 37 °C for 1.5h. After polymerization, the re-embedded gels were washed with ddH_2_O three times for 10 min each time and kept the samples in ddH_2_O until Ex-dSTORM imaging.

### Ex-dSTORM Imaging

A custom-modified imaging platform was assembled around a commercial inverted microscope (Eclipse Ti2-E, Nikon), integrated with an automated focus-locking system and a laser merge module (ILE, Spectral Applied Research) supporting independent control of four excitation lines. To enable uniform epi-illumination across expanded samples, laser beams at 637 nm (140 mW, OBIS 637 LX, Coherent), 561 nm (150 mW, Jive561, Cobolt), 488 nm (150 mW, OPSL 488 LX, Coherent), and 405 nm (100 mW, OBIS 405 LX, Coherent) were spatially homogenized using a Borealis Conditioning Unit (Spectral Applied Research). These beams were aligned and focused into the rear focal plane of a high numerical aperture oil-immersion objective (100×, NA 1.49, CFI Apo TIRF, Nikon) to achieve wide-field illumination suitable for single-molecule imaging.

Prior to imaging, re-embedded hydrogels were mounted in a custom-designed holder (Figure S14) and incubated twice in imaging buffer consisting of Tris–HCl and NaCl (TN buffer, pH 8.0), 10–100 mM mercaptoethylamine (MEA, pH 8.0), 10% glucose (G5767, Sigma-Aldrich), 0.5 mg/mL glucose oxidase, and 40 μg/mL catalase to promote fluorophore blinking. Both wide-field and super-resolution images were acquired. Wide-field imaging was performed under epifluorescence illumination. For Ex-dSTORM acquisition, high-power 637 and 561 nm lasers (∼3 kW cm^–2^) were used to switch the majority of Alexa Fluor 647 and CF568 fluorophores into a dark state. Reactivation was achieved with a 405 nm laser, while a 488 nm laser was periodically triggered every 800 frames to perform in situ drift correction. Fluorescence emission was detected using an EMCCD camera (pixel size: 83.5 nm). Typically, 20,000–30,000 frames were acquired per data set with an exposure time of 20 ms per frame. Real-time single-molecule localization was performed using the MetaMorph Super-Resolution Module (Molecular Devices).

### Drift Correction and Chromatic Abbreviation Correction

To achieve precise single-molecule localization, lateral drift induced by the imaging system was corrected postacquisition. Alexa Fluor 488-conjugated ATP synthase (1/100 dilution, ab109867, Abcam) was employed as a fiducial marker for drift tracking. Lateral displacement and fluorescence intensity were assessed using a custom plugin in ImageJ. Marker signal removal and drift correction were performed using a combination of LabVIEW, MATLAB, and ImageJ routines. Following alignment, image sequences were reconstructed in MetaMorph, and the resulting super-resolution images were enhanced using a Gaussian filter (0.75–1.2 pixels) in ImageJ (Figure S15). For two-color data sets, chromatic aberration was corrected using a custom code that spatially adjusted each pixel in the red fluorescence channel according to a predefined transformation function.

### Deformation Test

Blank gels were prepared by placing approximately 20 μL of gel solution into 1.5 mL Eppendorf tubes and polymerizing them at 37 °C. Following polymerization, the gels were immersed in deionized water to achieve full expansion. Deformation was assessed by calculating the ratio of the deformation radius to the original gel radius, as described in the TREx paper (Figure S3a).^23^

### Imaging Analysis

To assess centriole diameter, measurements were consistently taken at the distal region corresponding to the location of distal appendage proteins or distal lumen protein, using FBF1 or C2CD3 as a reference marker (Figure S16a). Diameters of centrioles and FBF1 were normalized by dividing by the average gel expansion factor specific to each method (4.3 for U-ExM, 9.6 for Magnify, and 8.3 for hiHomoExM). To evaluate expansion homogeneity between proteins within the same organelle, we calculated the diameter ratio of FBF1 to the distal centriole region from top-view images on a per-organelle basis (Figure S16b).

### Circularity Quantification

Circularity was quantified using a radial distribution-based metric. Briefly, individual protein clusters were identified to obtain the (*x*_*i*_,*y*_*i*_)­coordinates of each punctum. The centroid of each ring structure (*x*_0_,*y*_0_) was calculated as the mean position of all detected puncta:

$x_{0}=\frac{1}{n}\sum_{i=1}^{n}x_{i},y_{0}=\frac{1}{n}\sum_{i=1}^{n}y_{i}$ The radial distance of each punctum from the centroid was then computed as

$r_{i}=\sqrt{{\left(x_{i}-x_{0}\right)}^{2}+{\left(y_{i}-y_{0}\right)}^{2}}$ Circularity was quantified using a Circularity Index (*CI*) defined as

$CI=1-\frac{\sigma_{r}}{r_{mean}}$ where *r*_mean_ represents the mean radial distance and σ_*r*_ represents the standard deviation of the radial distances. This analysis was applied to U-ExM, hiHomoEx, and nonexpanded dSTORM data sets to allow direct comparison of structural circularity across imaging modalities.

### Statistics

The statistical analysis among different experiments was assessed by unpaired two-tailed *t*-test, one-way ANOVA test, or two-way ANOVA test using GraphPad Prism. Additionally, each data in this research was performed from at least two to three independent experiments.

## Supplementary Material



## Data Availability

All the data supporting the findings described this study are available within the article and Supporting Information and are available from the corresponding author upon reasonable request. All experiments were conducted at least three times, and representative images are shown for each experiment.

## Notes

The Supporting Information is available free of charge at Comparison of gel expansion factors; solubility test of sodium acrylate in the gel monomer solution; deformation assessment of expansion gel; circularity analysis of distal appendage proteins; centriole circularity under different orientations; hydrogel re-embedding procedure; expanded hydrogel re-embedding test; substructural organization of C2CD3 by hiHomoEx-dSTORM; hiHomoEx-dSTORM images of centriolar proteins; labeling efficiency test; comparison of centriole proximal ultrastructure; working time comparison of high-fold expansion microscopy methods; customized imaging holder; workflow of drift correction; image analysis; high-fold expansion microscopy comparison table; antibodies list, and hiHomoEx protocol. The Supporting Information is available free of charge at https://pubs.acs.org/doi/10.1021/acsnano.6c05501.
