# Supplementary material for: High-fold Homogeneous Expansion Microscopy Reveals Ultrastructural Centrioles
Source: ACS Nano. 2026 May 4;20(21):15716–29. doi: 10.1021/acsnano.6c05501 (PMC13235643; doi:10.1021/acsnano.6c05501)
Supplement: Supplementary file 1 [file nn6c05501_si_001.pdf]

Supporting Information

High-fold Homogeneous Expansion Microscopy Reveals Ultrastructural Centrioles

Wen-Qing Yang[†,‡,ª], Ting-Jui Ben Chang[†,‡,ª], Liang-Chen Pan[†, ‡], T. Tony Yang*[†, ‡]

[†]Department of Electrical Engineering, National Taiwan University, Taipei, 10617, Taiwan

[‡]Graduate Institute of Biomedical Electronics and Bioinformatics, National Taiwan University, Taipei, 10617, Taiwan

[ª]These authors contributed equally.

*Corresponding author:

T. Tony Yang

tonyyang@ntu.edu.tw

a

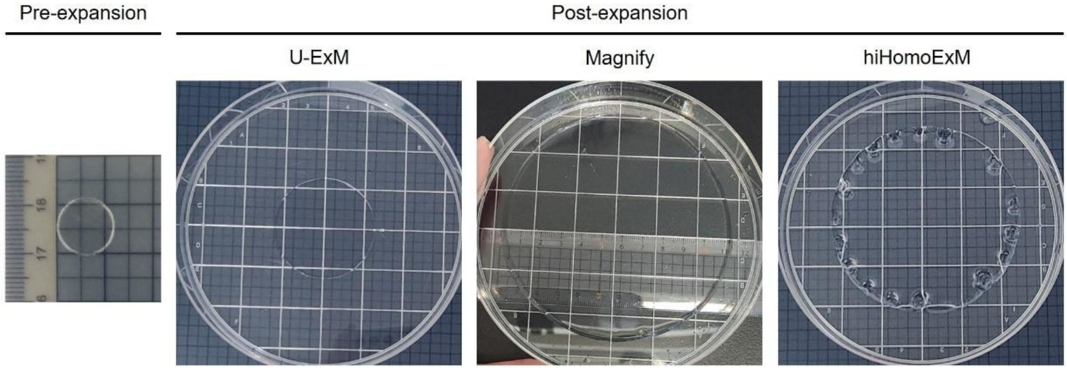

b

	Post-expansion		
	U-ExM	Magnify	hiHomoExM
Gel EF (Mean ± s.d.)	4.3 ± 0.03	9.6 ± 0.16	8.3 ± 0.16

Figure S1. Comparison of gel expansion factors: U-ExM, Magnify, and hiHomoExM.

a Representative images of pre-expansion and post-expansion gels for each method: U-ExM, Magnify, and hiHomoEx. **b** Quantification of the gel expansion factor (EF) for each method. Mean ± s.d. of gel EFs: 4.3 ± 0.03 (U-ExM, n = 4), 9.6 ± 0.16 (Magnify, n = 4), and 8.3 ± 0.16 (hiHomoEx, n = 4).

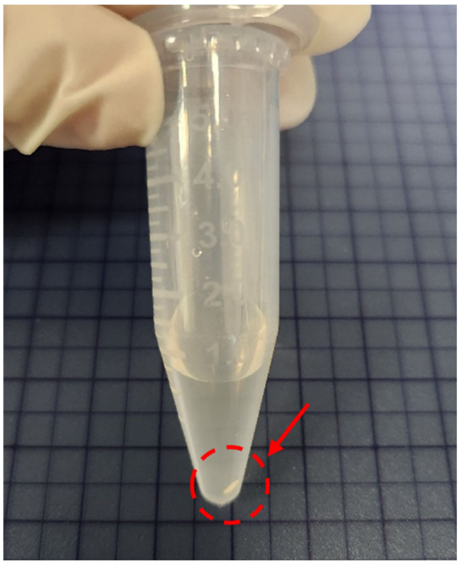

Figure S2. Oversaturation of sodium acrylate at high DMAA concentrations.

Image of a 16% DMAA monomer gel solution showing precipitation due to sodium acrylate oversaturation (red arrow).

a

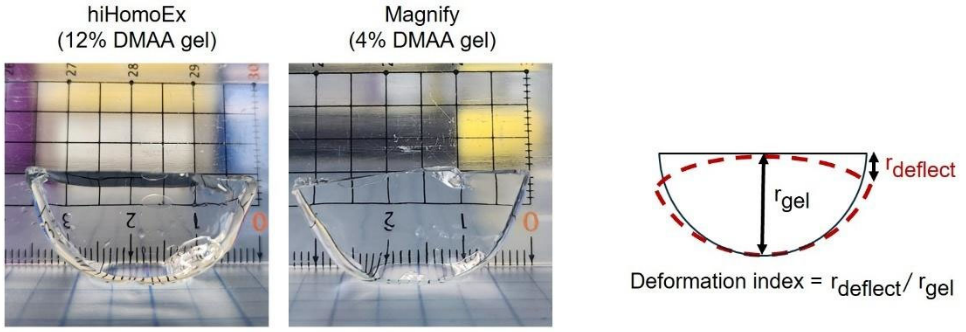

b

	hiHomoEx (12% DMAA gel)	Magnify (4% DMAA gel)
Deformation index (Mean ± s.d.)	0.018 ± 0.002 (N = 3)	0.065 ± 0.001 (N = 3)

Figure S3. Deformation assessment of expansion hydrogels at varying DMAA concentrations

a Representative gel images of the deformation test[1] for gels containing 12% DMAA and 4% DMAA. **b** Quantification of the deformation index for gels with two DMAA concentrations (12% and 4%). Mean ± s.d. of deformation index: 0.018 ± 0.002 (12% DMAA, hiHomoEx, N = 3) and 0.065 ± 0.001 (4% DMAA, Magnify, N = 3).

a

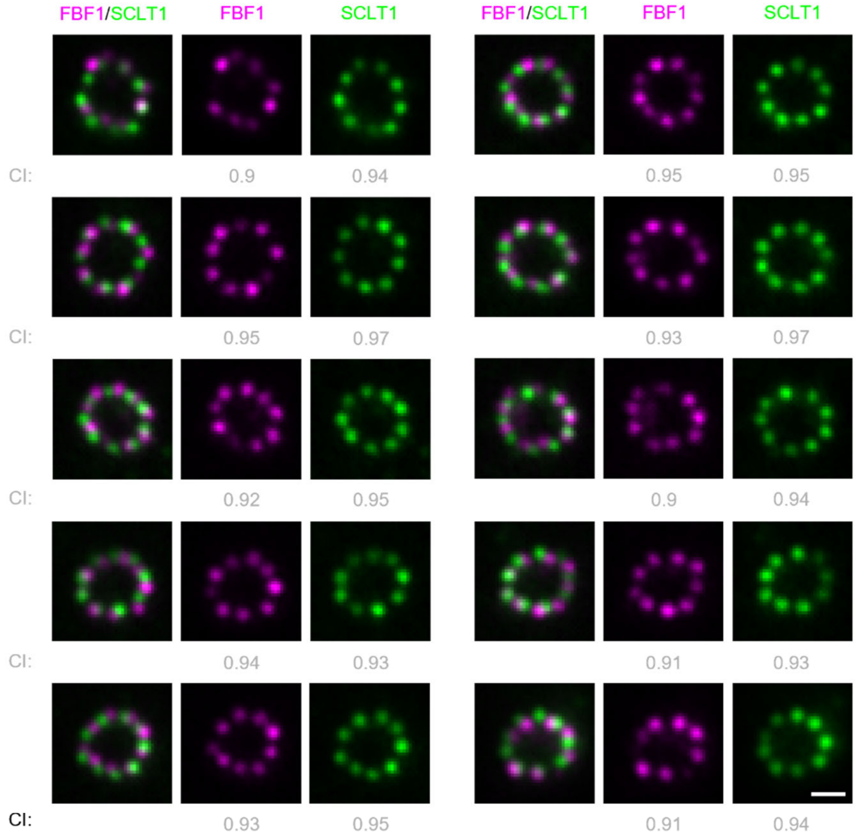

b

	SCLT1	FBF1
Circularity index (CI)	0.95 ± 0.01	0.92 ± 0.02

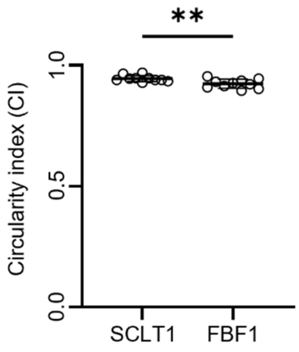

Figure S4. Distinct radial organization of distal appendage proteins revealed by circularity analysis.

a Two-color U-ExM images of SCLT1 (green; AF647) and FBF1 (magenta; CF568). Scale bar, 200 nm (biological scale). **b** Quantification of circularity index from (**a**), showing higher circularity for SCLT1 compared to FBF1. Statistical analysis was performed using unpaired two-tailed t-tests (** $p < 0.01$).

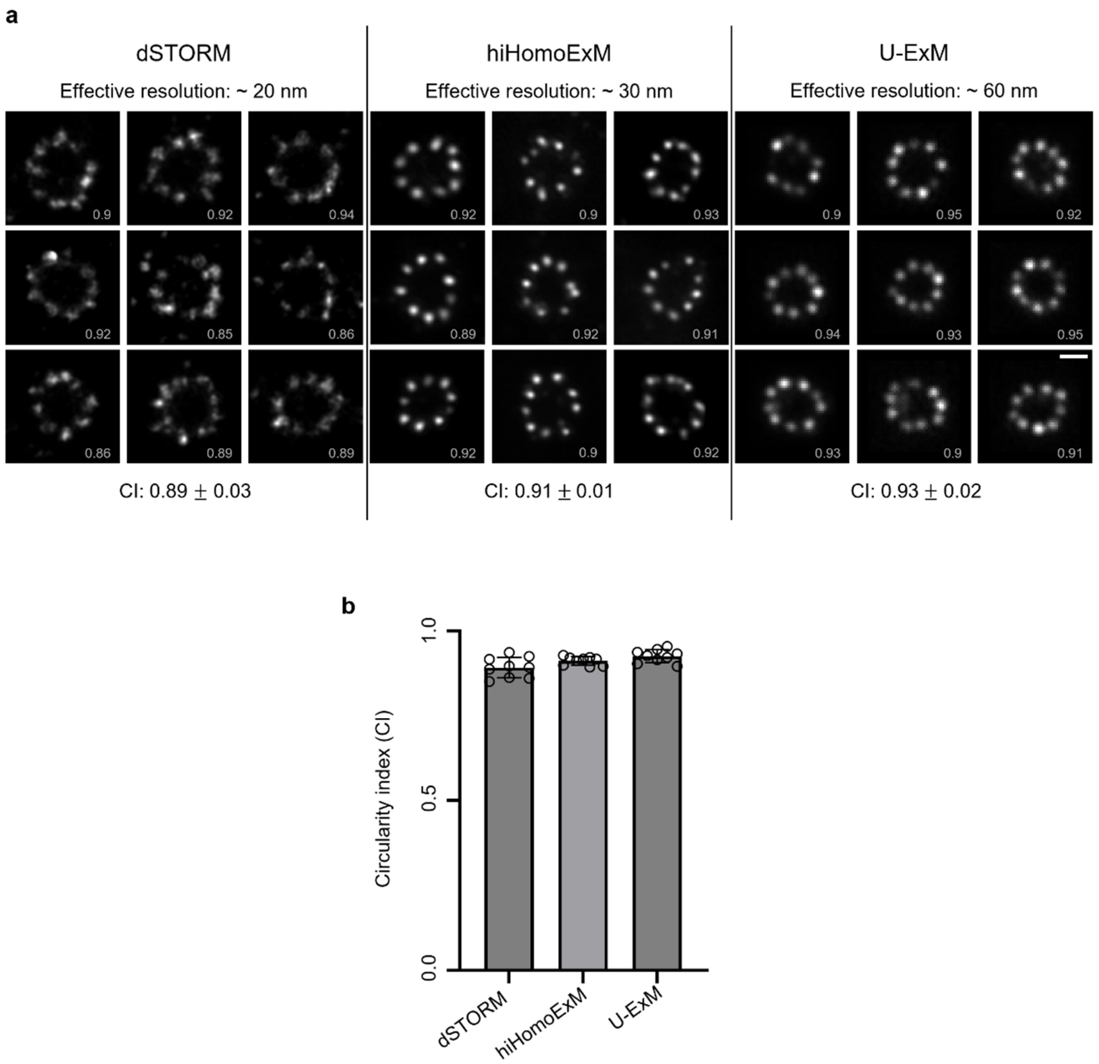

Figure S5. Resolution-dependent variation in circularity measurements of distal appendage structures.

a FBF1 images (grey; AF647 and CF568) acquired by dSTORM, hiHomoExM, and U-ExM, with corresponding circularity indices. Scale bar, 200 nm (biological scale). **b** Quantification of circularity index from (**a**), showing higher circularity values at lower effective resolution.

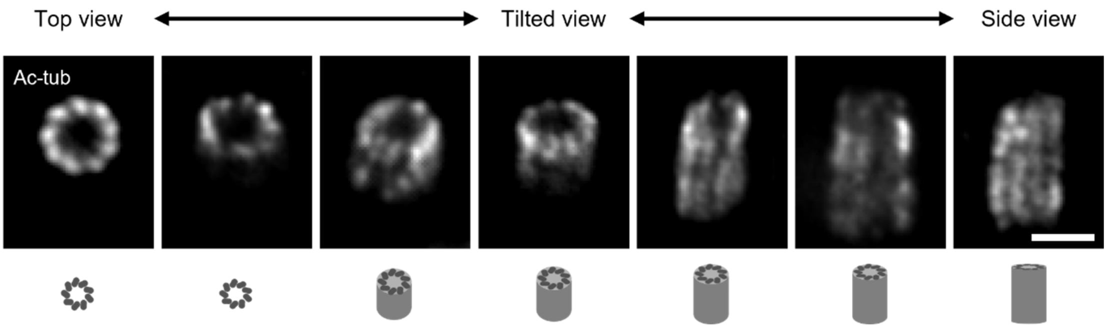

Figure S6. Apparent centriole circularity depends on orientation relative to the imaging plane.

Representative hiHomoExM images of centrioles labeled with Ac-tub (grey; CF568) from top, tilted, and side views, with cartoons below indicating orientation. Scale bar, 250 nm (biological scale).

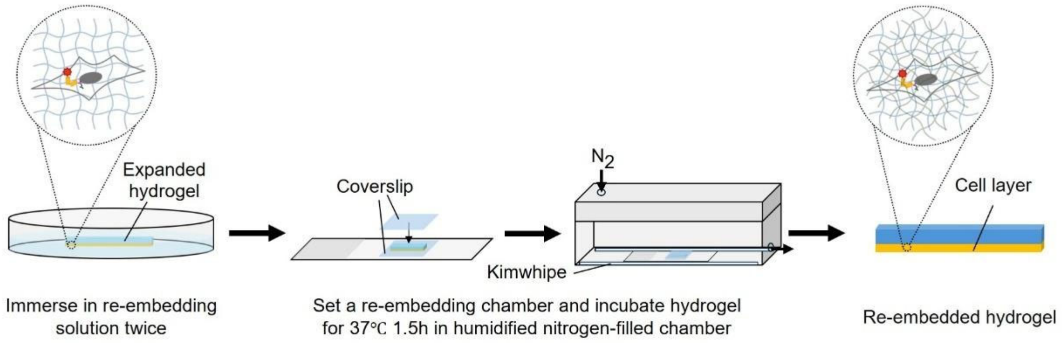

Figure S7. Schematic workflow of the expanded hydrogel re-embedding procedure.

During re-embedding, the expanded hydrogel is immersed in the re-embedding solution, allowing the solution to diffuse throughout the gel. The hydrogel is then incubated in a humidified, nitrogen-filled chamber to prevent oxygen inhibition of polymerization and to improve gel uniformity, ensuring more efficient and complete polymerization. Finally, the re-embedded hydrogel retains its expanded size during Ex-dSTORM imaging.

a

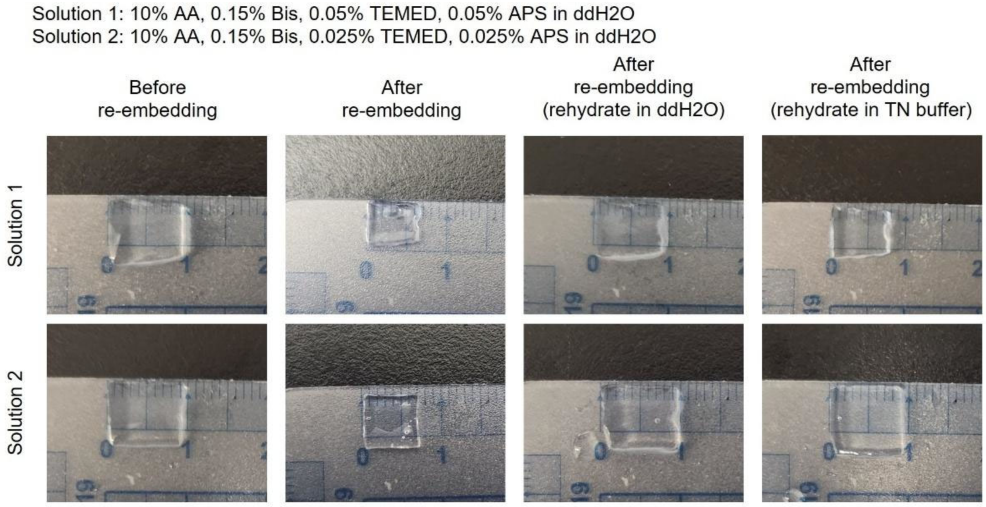

b

	Gel size (cm)			Retention rate
	Before re-embedding	After re-embedding		
		Rehydrate in ddH2O	Rehydrate in TN buffer	
Solution 1	1	0.91 ± 0.02	0.82 ± 0.02	82%
Solution 2	1	0.98 ± 0.02	0.96 ± 0.01	96%

Figure S8. Optimization of the re-embedding solution to preserve gel size during Ex-dSTORM imaging.

a Representative images of hydrogels before and after re-embedding, followed by rehydration in different solutions (ddH2O and TN buffer), to evaluate two re-embedding conditions by varying TEMED and APS concentrations (Solution 1 and Solution 2). **b** Quantification of gel size before and after re-embedding, followed by rehydration in ddH2O or TN buffer, using Solution 1 and Solution 2. Mean ± s.d. of gel size with Solution 1: 0.91 ± 0.02 cm (rehydrated in ddH2O, n = 3) and 0.82 ± 0.02 cm (rehydrated in TN buffer, n = 3). Mean ± s.d. of gel size with Solution 2: 0.98 ± 0.02 cm (rehydrated in ddH2O, n = 3) and 0.96 ± 0.01 cm (rehydrated in TN buffer, n = 3). Retention rate was calculated by dividing the gel size after re-embedding (rehydrated in TN buffer) by the gel size before re-embedding. Retention rates: 82% for Solution 1

and 96% for Solution 2.

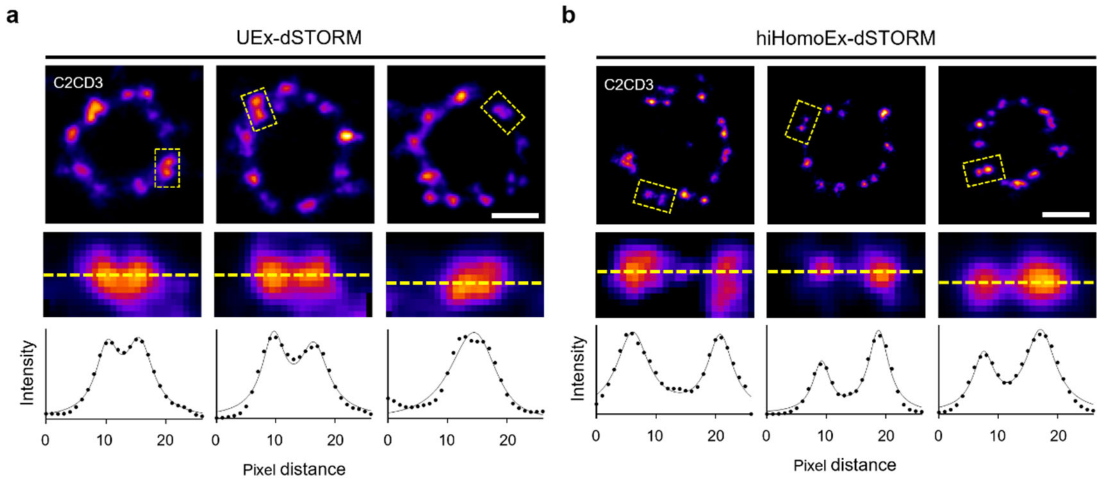

Figure S9. hiHomoEx-dSTORM reveals substructural organization of C2CD3.

a UEx-dSTORM images of C2CD3; yellow dashed boxes indicate regions where intensity profiles of individual clusters were measured. **b** hiHomoEx-dSTORM images of C2CD3; yellow dashed boxes indicate regions where intensity profiles of individual clusters were measured. Scale bar, 60 nm (a, b). All scale bars are shown in biological scale.

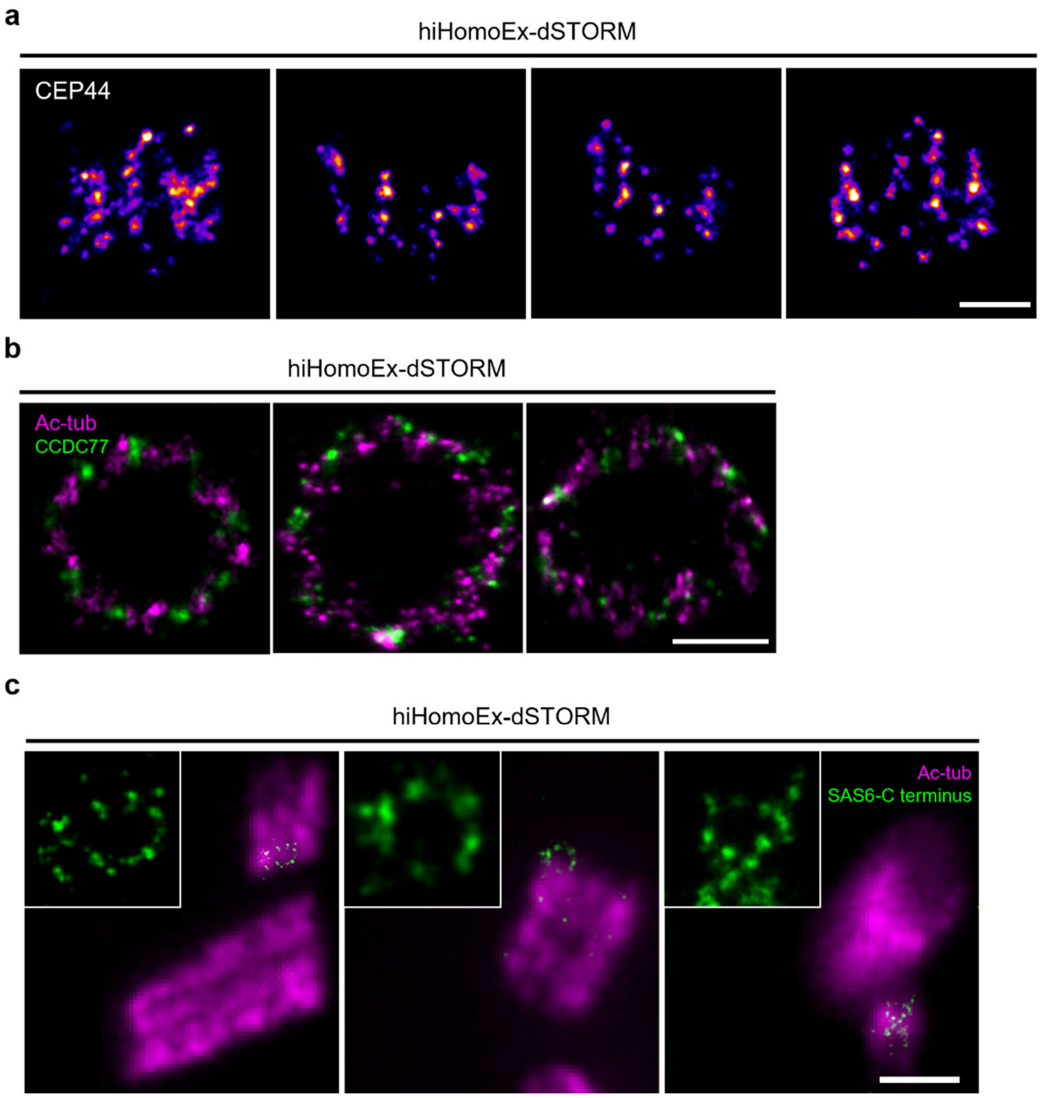

Figure S10. hiHomoEx-dSTORM images of centriolar proteins.

a Lateral-view hiHomoEx-dSTORM images of CEP44. Scale bar, 100 nm. **b** Two-color hiHomoEx-dSTORM images of Ac-tub (AF647; magenta) and CCDC77 (CF568; green) in top view. Scale bar, 100 nm. **c** hiHomoEx-dSTORM images of the SAS6 C-terminus (CF568; green) from a tilted view, merged with a widefield image of acetylated tubulin (Ac-tub, AF647; magenta). Scale bar, 200 nm. All scale bars are shown in biological scale.

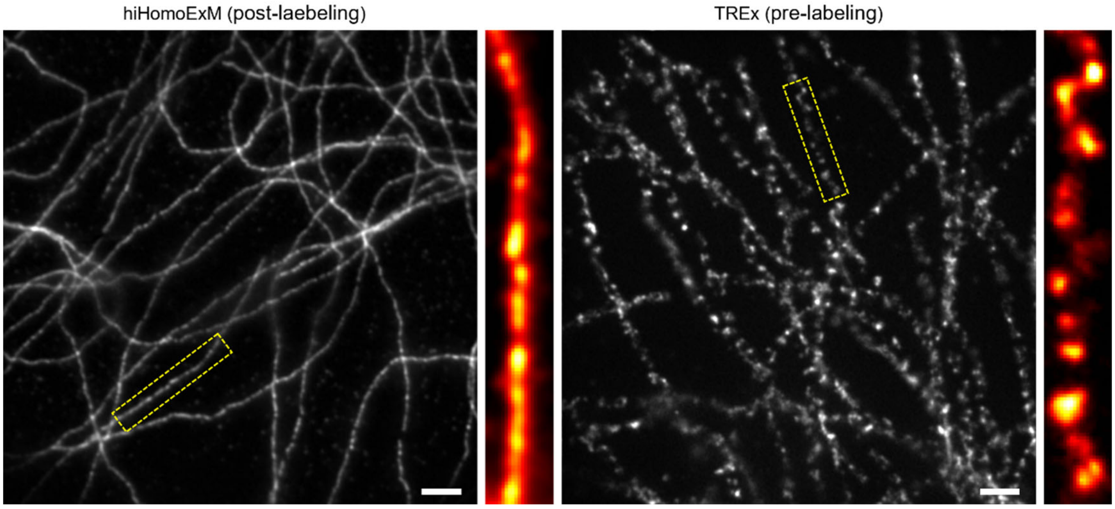

Figure S11. Comparison of microtubule labeling efficiency using post- and pre-expansion labeling strategies.

Representative images of microtubules labeled with alpha-tubulin (grey; CF568) using hiHomoExM (post-labeling) and TREx (pre-labeling). Yellow dashed boxes indicate the regions shown in the zoom-in images. Scale bar, 500 nm. All scale bars are shown in biological scale.

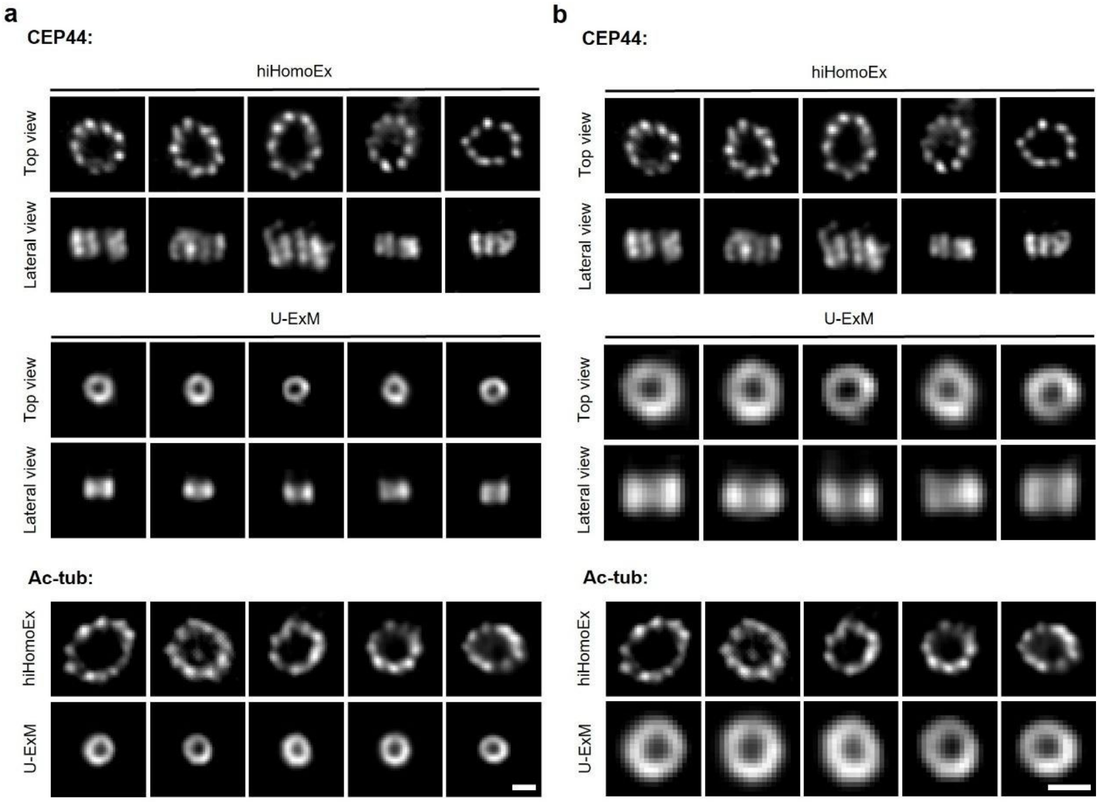

c

	CEP44		Ac-tub (centriole proximal end)	
	Original scale (nm)	Biological scale (nm)	Original scale (nm)	Biological scale (nm)
hiHomoEx	2131.2 ± 204.3	247.8 ± 23.8	2245.1 ± 161.3	261.0 ± 18.8
U-ExM	1043.3 ± 80.0	231.8 ± 17.8	1100.0 ± 72.9	244.4 ± 16.2

Figure S12. Comparative ultrastructural visualization of the centriole proximal region using hiHomoEx and U-ExM.

a Comparative widefield images of CEP44 and Ac-tub in the proximal centriole region acquired with hiHomoEx and U-ExM, shown at original scale. Scale bar, 1 μm (non-corrected). **b** Corresponding images in (**a**) displayed at biological scale to reflect true structural dimensions. Scale bar, 200 nm (corrected). **c** Quantitative comparison of CEP44 and Ac-tub diameters based on images in (**a**) and (**b**), presented with both original and biological scales. Mean ± s.d. of CEP44 diameter: 2131.2 ± 204.3 nm

(hiHomoEx, original scale, n = 10), 1043.3 ± 80 nm (U-ExM, original scale, n = 10), 247.8 ± 23.8 nm (hiHomoEx, biological scale, n = 10), 231.8 ± 17.8 nm (U-ExM, biological scale, n = 10). Mean ± s.d. of Ac-tub diameter: 2245.1 ± 161.3 nm (hiHomoEx, original scale, n = 5), 1100 ± 72.9 nm (U-ExM, original scale, n = 5), 261 ± 18.8 nm (hiHomoEx, biological scale, n = 5), 244.4 ± 16.2 nm (U-ExM, biological scale, n = 5).

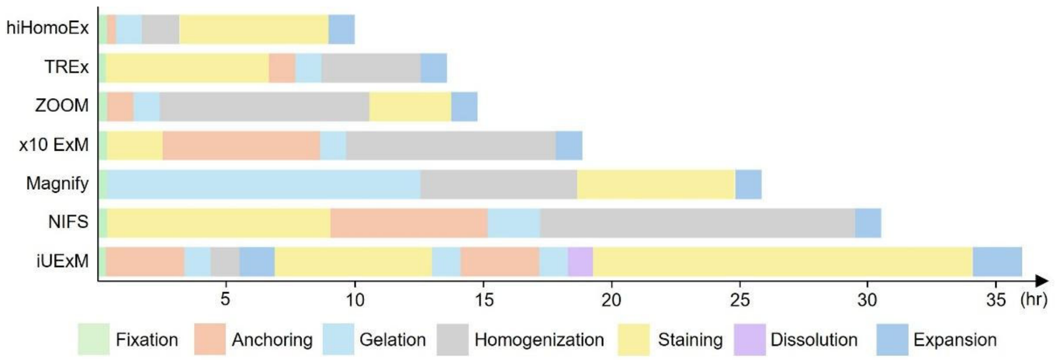

Figure S13. Working time comparison of high-fold expansion microscopy methods.

Comparison of total workflow duration and step-by-step time requirements across various high-fold expansion microscopy methods—including hiHomoEx, TREx[1], ZOOM[2], X10 ExM[3], Magnify[4], NIFS[5], and iUExM[6]—applied to samples cultured cells.

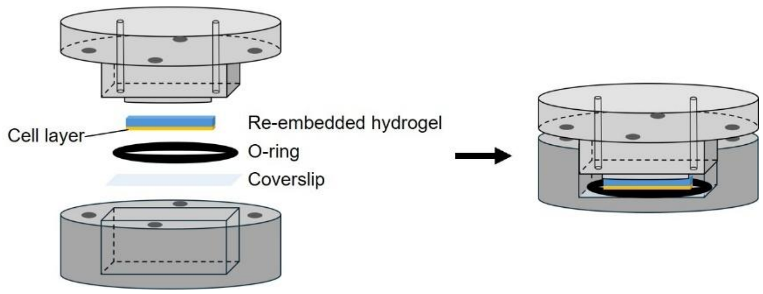

Figure S14. Schematic diagram of the customized holder for Ex-dSTORM imaging.

The re-embedded hydrogel is sandwiched between a coverslip and the upper lid of the holder to stabilize the sample during image acquisition. Prior to Ex-dSTORM imaging, imaging buffer is added through two holes on the upper lid. The confined space of the holder minimizes oxidative reactions caused by air exposure, providing an optimal environment for the imaging buffer to interact with the fluorophores.

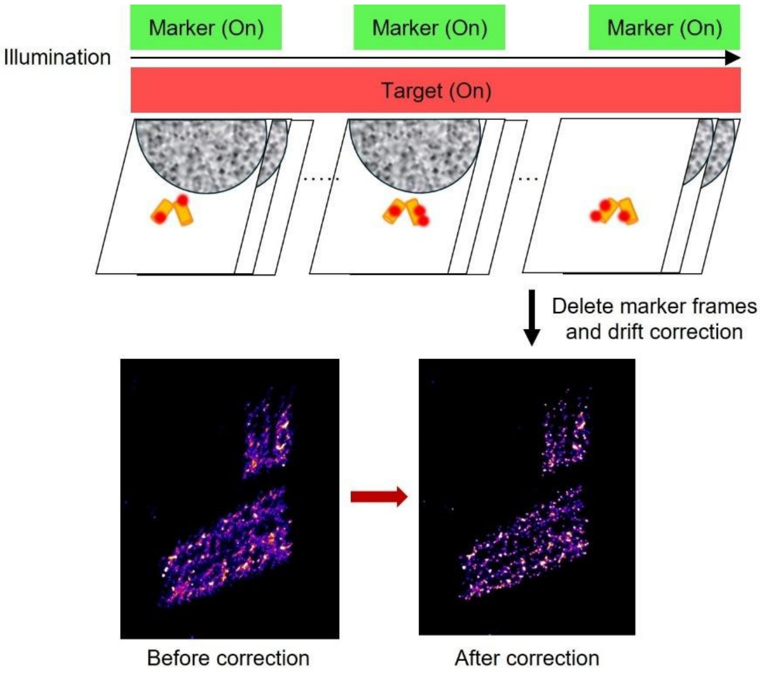

Figure S15. Schematic workflow of in-situ drift correction.

During Ex-dSTORM imaging, the in-situ marker (ATP synthase-AF488) is periodically illuminated every 800 frames, while the target channel (AF647 or CF568-fluorophores capable of triggering the blinking mechanism) is illuminated continuously. After image acquisition, drift correction is performed using the in-situ marker, and the corresponding marker frames are removed. Finally, all corrected frames are overlaid to reconstruct the target structure.

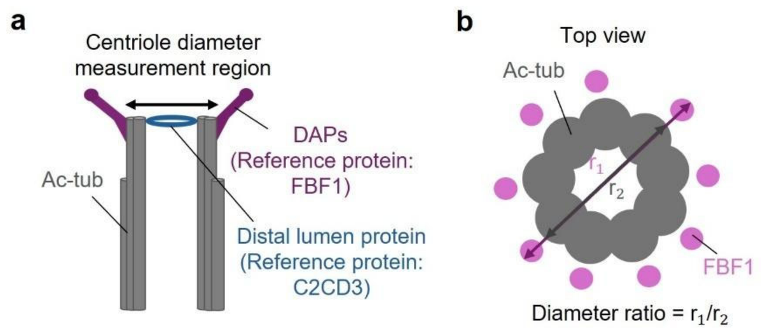

Figure S16. Schematic diagram of image analysis.

a Schematic diagram of centriole diameter measurement. Centriole diameters were measured consistently in the distal region. FBF1 (a distal appendage protein) or C2CD3 (a distal lumen protein) was used as a reference marker to indicate the distal region of the centriole. **b** Schematic diagram of homogeneity analysis using two different proteins on the same organelle. The diameter ratio was calculated by dividing the FBF1 diameter (r_1) by the distal centriole diameter (r_2), serving as an indicator of expansion homogeneity at the ultrastructural level.

Table S1 Workflow duration, stepwise timing, and claimed expansion factors of high-fold expansion microscopy methods in cultured cells.

High-fold ExM	Labeling strategy	Workflow	Total time	Expansion factor
hiHomoEx	Post-labeling	Fixation (7-10min) → Anchoring (7-10min) → Gelation (1h) → Homogenization (denaturation, 1.5h) → Staining (6h) → Expansion (1h)	~10 h	~8-9x
Magnify	Post-labeling	Fixation (10min) → Gelation (o/n) → Homogenization (denaturation, 6h) → Staining (6h) → Expansion (1h)	~25 h	~8-11x
ZOOM	Post-labeling	Fixation (10min) → Anchoring (1h) → Gelation (1h) → Hydrolysis (8h) → Staining (3.5h) → Expansion (1h)	~14.5 h	~10x
X10 ExM	Pre-labeling	Fixation (10min) → Staining (2.5h) → Anchoring (6h) → Gelation (1h) → Homogenization (digestion, 8h) → Expansion (1h)	~18.5 h	~10x
TREx	Pre-labeling	Fixation (10min) → Staining (7h) → Anchoring (1h) → Gelation (1h) → Homogenization (denaturation, 3h/ digestion, 4h) → Expansion (1h)	~13-14 h	~10x
NIFS	Pre-labeling	Fixation (10min) → Staining (8.5h) → Anchoring (6h) → Gelation (2h) → Homogenization (digestion, o/n) → Expansion (1h)	~30 h	~9x
iUExM	Intermediate and post-labeling	Fixation (7-10min) → Anchoring (3h) → Gelation (1h) → Homogenization (denaturation, 1.5h) → 1st Expansion (1h) → Intermediate staining (6h) → Neutral gel embedding (1.5h) → Anchoring (3h) → 2nd monomer gel embedding (1.5h) → Dissolution (1h) → post-staining (24h) → Final expansion (o/n)	~2-3 day	~13-16x

Table S2 List of primary antibodies in this study.

Name of antibody	Host	Company	Cat no.	Dilution
Acetyl-alpha Tubulin (Ac-Tub, 6-11-b-1)	mouse IgG	Thermo Fisher	32-2700	1/200 - 1/100
Acetyl-alpha Tubulin (Lys40)	rabbit IgG	Cell signaling	5335S	1/200 - 1/100
FBF1	rabbit IgG	Proteintech	11531-1-AP	1/200 - 1/100
C2CD3	rabbit IgG	Sigma-Aldrich	HPA040433	1/100
ODF2-C	rabbit IgG	Abcam	ab43840	1/100
MNR (KIAA0753)	rabbit IgG	Novus Biologicals	NBP1-90929	1/100
CEP90 (PIBF1)	rabbit IgG	Proteintech	14413-1-AP	1/100
OFD1	rabbit IgG	Sigma-Aldrich	HPA031103	1/100
CEP128	rabbit IgG	Abcam	ab118797	1/100
CP110	rabbit IgG	Proteintech	12780-1-AP	1/100
CEP44	rabbit IgG	Proteintech	24457-1-AP	1/100
CCDC77	rabbit IgG	Proteintech	26369-1-AP	1/100
SAS6	mouse IgG2b	Santa Cruz	sc-81431	1/100
ATP synthase	mouse IgG	Abcam	ab109867	1/100

Alpha-tubulin	mouse IgG1	Santa Cruz	sc-32293	1/200 - 1/100
Alpha-tubulin	rat IgG	Abcam	ab6160	1/100
GM130	mouse IgG	Abcam	ab169276	1/100
TOMM20	rabbit IgG	Abcam	ab186735	1/100
NUP98-96	rabbit IgG	Proteintech	12329-1-AP	1/100
GFP	rabbit IgG	Abcam	ab290	1/100

Table S3 hiHomoExM preparation protocol

Procedure	Reagents	Working condition
Fixation	MeOH (centriolar proteins, microtubules, Golgi apparatus)<br>3%PFA + 0.1%GA (mitochondria)	Pre-cold MeOH: -20°C 7 min<br>3%PFA + 0.1%GA: 10 min at RT
	Pre-extraction (nuclear pore complex):<br>2.4% PFA<br>0.1% PBS-Triton<br>0.2% PBS-Triton<br>0.4% PBS-Triton<br>1x PBS	2.4% PFA 30 s → 0.1% or 0.4% PBS-Triton 3 min → 1x PBS wash twice (each 5 min) → 2.4% PFA 20 min → 1x PBS wash twice (each 5 min) → 0.2% PBS-Triton 10 min →1x PBS wash twice (each 5 min)
Anchoring	Anchoring solution:<br>0.1% methacrolein in 1x PBS	37°C 7-10 min
Gelation	Gel solution:<br>12% (v/v) DMAA<br>34% (w/v) SA<br>10% (w/v) AA<br>0.0001% (w/v) Bis<br>1% (w/v) NaCl<br>1x PBS<br>0.15% (w/v) APS<br>0.15% (w/v) TEMED<br>ddH_2O	37°C 1 h
Denaturation	Denaturation buffer:<br>200mM SDS<br>200mM NaCl<br>50mM Tris (pH6.8)	85°C 1.5 h
Expansion	ddH_2O	Exchange ddH_2O at least 5 times (each 10 min), until 8x expansion

Reference

(1) Damstra, H. G. J.; Mohar, B.; Eddison, M.; Akhmanova, A.; Kapitein, L. C.; Tillberg, P. W. Visualizing cellular and tissue ultrastructure using Ten-fold Robust Expansion Microscopy (TREx). *eLife* **2022**, *11*, e73775. DOI: 10.7554/eLife.73775.

(2) Park, H.-E.; Choi, D.; Park, J. S.; Sim, C.; Park, S.; Kang, S.; Yim, H.; Lee, M.; Kim, J.; Pac, J.; et al. Scalable and Isotropic Expansion of Tissues with Simply Tunable Expansion Ratio. *Advanced Science* **2019**, *6* (22), 1901673. DOI: https://doi.org/10.1002/advs.201901673.

(3) Truckenbrodt, S.; Sommer, C.; Rizzoli, S. O.; Danzl, J. G. A practical guide to optimization in X10 expansion microscopy. *Nature Protocols* **2019**, *14* (3), 832–863. DOI: 10.1038/s41596-018-0117-3.

(4) Klimas, A.; Gallagher, B. R.; Wijesekara, P.; Fekir, S.; DiBernardo, E. F.; Cheng, Z.; Stolz, D. B.; Cambi, F.; Watkins, S. C.; Brody, S. L.; et al. Magnify is a universal molecular anchoring strategy for expansion microscopy. *Nature Biotechnology* **2023**, *41* (6), 858–869. DOI: 10.1038/s41587-022-01546-1.

(5) Li, H.; Warden, A. R.; He, J.; Shen, G.; Ding, X. Expansion microscopy with ninefold swelling (NIFS) hydrogel permits cellular ultrastructure imaging on conventional microscope. *Science Advances 8* (18), eabm4006. DOI: 10.1126/sciadv.abm4006.

(6) Louvel, V.; Haase, R.; Mercey, O.; Laporte, M. H.; Eloy, T.; Baudrier, É.; Fortun, D.; Soldati-Favre, D.; Hamel, V.; Guichard, P. iU-ExM: nanoscopy of organelles and tissues with iterative ultrastructure expansion microscopy. *Nature Communications* **2023**, *14* (1), 7893. DOI: 10.1038/s41467-023-43582-8.
